# Overview of styles, content, learning effects and attitudes of students towards digitally enhanced physiotherapy education – a scoping review

**DOI:** 10.1186/s12909-025-06750-6

**Published:** 2025-02-04

**Authors:** Larissa Pagels, Oskar Schindler, Kerstin Luedtke

**Affiliations:** 1 Institute of Health Sciences, Department of Physiotherapy, Pain and Exercise Research Luebeck (P.E.R.L.), University of Luebeck, Ratzeburger Allee 160, 23562 Luebeck, Germany; 2 DHGS Deutsche Hochschule für Gesundheit und Sport, Berlin, Germany; 3 Center of Brain, Behavior and Metabolism (CBBM), University of Luebeck, Luebeck, Germany

**Keywords:** Digital, Education, Physiotherapy, Hybrid, Learning

## Abstract

**Background:**

Digital competences are essential for lifelong learning, as highlighted by the European Commission and emphasized in the Digital Education Action Plan 2021–2027. The COVID-19 pandemic necessitated an unprecedented shift to online education, profoundly impacting fields like physiotherapy that heavily rely on practical skills. This scoping review aims to provide an overview of currently applied digitally enhanced learning methods, content, effect on knowledge gain and student perceptions in physiotherapy education.

**Methods:**

Following PRISMA guidelines for scoping reviews, a comprehensive search was conducted across multiple databases, including Medline, Web of Science, and ERIC, incorporating hand searches and expert consultations. Studies were included if they reported on any digitally enhanced educational methods in physiotherapy education, involving qualitative studies, clinical trials, observational studies, or case reports published in English or German from 2010 to February 2024. Data extraction focused on the digital tools that were used, the educational contents, individually measured outcomes, and the impact of digital education.

**Results:**

Out of 2988 screened studies, 67 met the inclusion criteria, encompassing 7160 participants. These sources of evidence primarily used quantitative methods (*n* = 51), with a minority using qualitative (*n* = 7) or mixed methods (*n* = 6). Nearly half employed hybrid educational approaches. Outcome measures included knowledge, performance, perception, satisfaction, and attitudes. Most sources of evidence reported positive impacts of digitally enhanced education, particularly in knowledge transfer and skill performance. Synchronous and asynchronous methods were used, with varying success across theoretical and practical courses. Gamification and virtual reality emerged as promising tools for enhancing engagement and learning outcomes. However, challenges included the limited direct interaction and perceived self-efficacy among students.

**Conclusion:**

Digitally enhanced learning formats in physiotherapy education can enhance learning experiences and is generally welcomed by students, especially when blended with traditional methods. The integration of innovative digital strategies holds promise for the future of physiotherapy training, contingent on comprehensive support and training for educators and students alike.

**Supplementary Information:**

The online version contains supplementary material available at 10.1186/s12909-025-06750-6.

## Introduction

Digital competences are stated as one of the key requirements for lifelong learning by the European Commission [1, 2]. The enhancement of these are prioritized in the European Commission Digital Education Action Plan 2021–2027, involving the ability to use digital technologies confidently, critically and responsibly and engage with them for learning, work and for participation in society [1, 3]. There appears to be increasing attention to the pedagogical use of digital technologies in higher education, but the possibilities are far from being fully exhausted [4]. For example, virtual reality (VR) and augmented reality (AR) represent innovative tools with significant potential in the education of healthcare providers, yet their full range of application in higher education remains underexplored.

Educational institutions worldwide were facing major challenges due to the pandemic caused by the Sars-COVID-19 virus. Where comprehensive containment strategies for digital / distance learning were not enforced by governments, students were unable to attend their usual classes [5, 6]. The switch to a purely online format at the beginning of 2020 was unprecedented [6–8]. This resulted in teachers being forced to switch from traditional face-to-face teaching to online teaching and making use of different e-learning methods to impart knowledge [9, 10]. In response to the pandemic lockdowns, educators faced the challenge of rapidly transitioning their curricula to fully online formats, particularly for content requiring face-to-face components. Teaching and assessing practical skills remotely posed significant difficulties [11, 12].

In degrees that offer theoretical and practical content such as physiotherapy, the limitations of distance learning for active skill acquisition (observation, palpation, examination, manipulative treatment, exercise techniques) may affect the perceived ability to gain knowledge and skill performance, further affecting students’ attitudes towards distance learning [13].

Student satisfaction with learning methods is closely linked to their overall knowledge acquisition and academic performance [14]. Hence, student satisfaction impacts motivation, engagement, and learning outcomes by creating a positive learning environment where students feel supported and encouraged to learn effectively [14–16].

There are several ways to implement digital learning tools (e.g., apps) and methods (e.g., flipped classroom) into physiotherapy education and thereby digitally enhance the education in physiotherapy degrees.

Veneri et al. (2011) reported on the role of computer-assisted learning in physiotherapy education in a systematic review, followed by Mącznik et al. (2015) who concluded that the use of technologies may enhance practical skills performance, knowledge acquisition and the development of critical and reflective thinking in physiotherapy students [17, 18]. More recently, Ødegaard et al. (2021) published a systematic review about the effectiveness of different digital learning designs in physiotherapy education, concluding that digital learning designs in the form of blended learning and distance learning were equally or more effective compared to traditional teaching [19].

A recent qualitative study about the characteristics of physiotherapy students’ digital learning practices shows that physiotherapy students adapt to, use and have experience with digital technologies in different learning contexts [20]. Furthermore, the development of informal digital learning practices (e.g., social media, online communities, and forums) was perceived as collaborative by the students. But these practices do not align with competence dimensions in the students’ study programs and students find it challenging to connect their informal digital learning practices with the practical skills and expectations required in clinical workspaces [20].

Recent studies analyzed the effect of rapidly changed curricula from face-to-face to digital education facing the pandemic restrictions [6, 21–23]. Online instruction in entry-level physiotherapy appears to be a viable option to address the changes due to the COVID-19 pandemic as it satisfies students and results in performance similar to in-person courses [6, 21–23]. The asynchronous remote training and feedback model, asynchronous seminars and blended learning courses have been found to be effective strategies for skill performance development in students and represent feasible alternatives to face-to-face training [24–26]. While the transition to online and hybrid teaching methods was necessitated by the Sars-COVID-19 pandemic, their continued implementation should depend on demonstrable benefits to student outcomes.

The aim of this scoping review was to summarize the effects of digitally enhanced educational methods in physiotherapy education on knowledge gain, skill performance, and student satisfaction as reported in the existing literature. Given the rapid growth of digitally enhanced learning since the COVID-19 pandemic, this review also provides an updated overview of teaching and learning methods, including those introduced or widely adopted after 2020. Furthermore, the review seeks to identify the most suitable digital learning methods for specific content areas and explore strategies to maintain student engagement, thereby building on insights from previously published reviews.

## Methods

The PRISMA recommendations were used for the design, conduction and report of this scoping review [27]. Furthermore, the methods were conducted in alignment with the JBI protocol [28]. The review protocol was pre-registered in the open science framework platform (OSF; 10.17605/OSF.IO/D6ECM).

The search was conducted in the databases Medline (PubMed), Web of Science, EBSCO Teacher Reference Center, ERIC Institute of Education Sciences, Fachportal Pädagogik with predefined combinations of MeSH terms and free-text terms and tested to identify relevant data of the research topic (Table 1). Hand searches were added by following the reference lists of the included studies.

**Table 1:** Combination of search terms for the data base search

Terms	Identifier
physiotherapy	1
education	2
digital OR hybrid OR virtual OR augmented OR flipped OR blended OR e-learning OR web-based OR streaming OR “distance education” OR “massive open online course” OR technology OR multimedia OR “user training” OR applications OR interactive OR gaming	3
**Database**	**Simplified strategy**
PubMed	1 AND 2 AND 3

The database results were entered into the online software Rayyan. First, titles and abstracts were screened by two independent reviewers against the eligibility criteria. All studies rated as eligible by at least one reviewer were screened in a full-text evaluation against the same eligibility criteria to ensure the comprehensive inclusion of relevant studies. The decisions for inclusion or exclusion of studies were made by two independent researchers in the abstract and full-text screening phase (LP, OS). Conflicting results were rated by a third independent researcher (KL).

Studies were included if they were clinical trials, observational studies or case reports and reported any kind of digitally enhanced educational method (hybrid, completely digital) in physiotherapy education. Hybrid education encompasses various models that integrate both remote and face-to-face learning. This approach also includes in-person instruction supplemented with digital tools (e.g., applications, augmented reality, and other technologies). It is often referred to as blended learning or digitally enhanced learning. These terms reflect the use of technology to complement traditional classroom methods [29]. Both quantitative and qualitative study designs were included. As this review focused on assessing both the direct effects on knowledge gain and performance improvement and secondary outcomes (e.g., student satisfaction, perception) of digitally enhanced education, either or both were required to be reported in the included studies. The study population had to be physiotherapy students with no limit of degree or year of studying. Only studies published in German or English language until February 2024 were included. Studies published before 2010 were excluded because there were limited uses of digital learning designs in physiotherapy education [19]. Studies were excluded if reporting on mixed health profession students and results for the physiotherapy students alone could not be distinguished.

Data from included studies were mapped in a pre-designed data extraction table. These included the number of participants, study inclusion and exclusion criteria, the form of education (e.g. augmented reality simulation, blended learning, open online course), the detailed intervention with information about how and how long the digital educational methods were applied, outcome measurements used to measure the effect of the digital education including soft outcomes (e.g. satisfaction, perception) and if there was a positive impact of digital education (Table I, supplementary material). Digital education was considered to have a positive impact on the outcome measures if studies reported improvements such as knowledge gain, enhanced skill performance, or increased student satisfaction with the teaching methods, directly attributable to the digital education intervention. Additionally, it was recorded whether the digital education was conducted due to the Sars-COVID-19 pandemic. The data extraction was performed by two researchers and counterchecked for correctness (LP, OS). Qualitative outcomes were analyzed by applying a narrative synthesis and summarized in a Sankey diagram. If data was not reported in the full-texts, authors were contacted via email and reminded after one week.

## Results

**Fig. 1:**
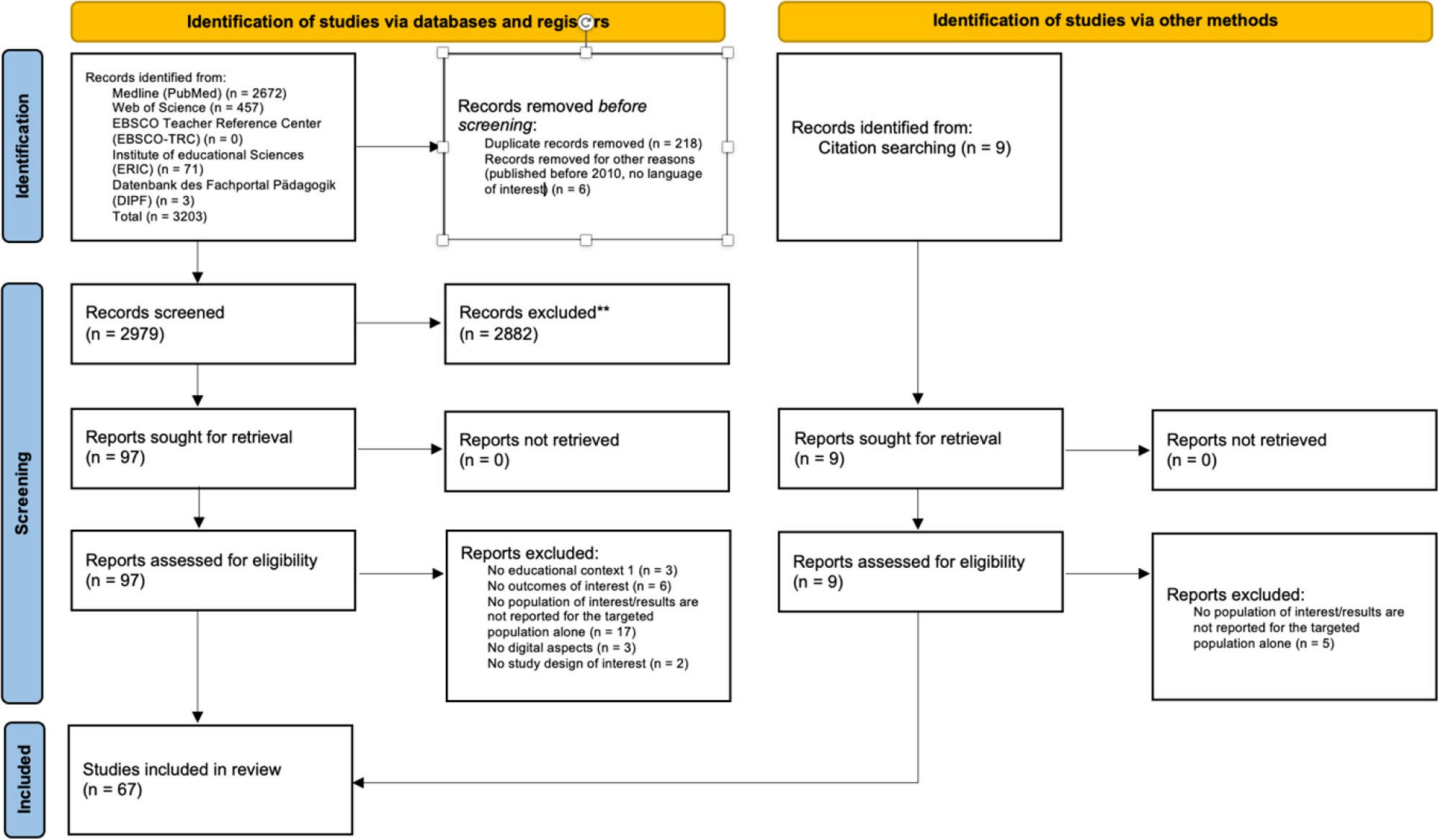
: Selection of sources of evidence. From: Page MJ, McKenzie JE, Bossuyt PM, Boutron I, Hoffmann TC, Mulrow CD, et al. The PRISMA 2020 statement: an updated guideline for reporting systematic reviews. BMJ 2021;372:n71. doi: 10.1136/bmj.n71

A total of 2988 studies were screened at title and abstract level. In the next step, the full texts of 106 studies were examined and subsequently, 67 sources of evidence were included into this review (Fig. 1).

### Study characteristics

**Fig. 2:**
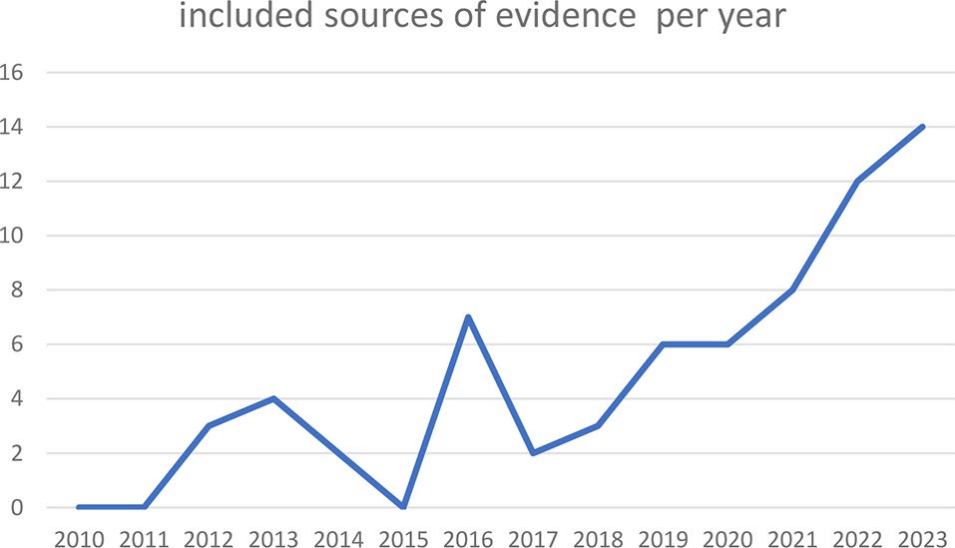
Overview of the number of publications per year

Included sources of evidence were published between 2012 and 2023 (Fig. 2), reporting on a total of 7160 participants (6514 physiotherapy students). Interestingly, no study was identified in 2015 (Fig. 2). Four sources of evidence also included physiotherapy professors [30], medical students [31], exercise physiology and exercise science students [32], students specialized in home care or elderly care and first and emergency aid students [33]. Only results for the physiotherapy students were included in this review. Most sources of evidence used a quantitative research approach for the purpose of analyzing the effect of digital or hybrid education (*n* = 54 (80%)), while 7 sources of evidence (10.4%) had a qualitative study design and 6 (9%) used a mixed methods approach. 47.8% (*n* = 32) of the sources of evidence reported hybrid educational methods [14, 21, 24, 25, 31, 32, 34–59] defined as digitally enhanced education, while the remaining 35 (52.2%) reported digital educational methods without any face-to-face sessions. The outcomes measured were either theoretical knowledge gain [14, 22, 25, 30, 34, 35, 37, 46, 49, 58–64] or practical knowledge gain and improvement in skill performance [6, 12, 14, 24–26, 30–32, 34–36, 38–43, 46, 49–51, 53–59, 62, 63, 65–79], perception [8, 12–14, 23–25, 35–37, 41, 43, 45, 48, 49, 52, 53, 55, 59, 62, 64, 66, 70, 74, 75, 77, 78, 80, 81], satisfaction [6, 14, 21–23, 35–38, 40, 44, 49, 55, 56, 62, 64, 67, 68, 70, 72, 74, 77, 78] or attitude [13, 21, 26, 34, 50] of the students towards the digital learning methods used in the respective sources of evidence. Other outcomes measured were the student’s opinion (e.g., relevance and comprehensibility of the resources, appropriateness of the content delivery method, quality of content, if digital learning is superior/inferior to traditional learning) about the digital education method used [8, 20, 23, 30, 33–35, 38, 39, 47, 48, 51, 52, 58, 60, 62, 63, 80–83] and perceived self-efficacy while learning in a digitally (enhanced) environment [26, 44, 73]. The Sars-COVID-19 pandemic was reported as a major reason for the conduction of the analyzed digital educational approach in 25 (37.3%) of the included sources of evidence [6, 8, 12, 13, 20–26, 33, 36, 37, 48, 50, 51, 54, 56, 57, 68, 69, 73, 79, 84]. The results of the individual sources of evidence are shown in Table I (supplementary material).

### Content delivery methods

The included sources of evidence used a variety of different delivery methods for the digitally enhanced teaching of physiotherapy students. In some cases, lectures that were originally developed for face-to face teaching were reorganized and held as synchronous online lecture [6, 8, 25, 31, 38, 43, 56–58, 62, 67, 81] or pre-recorded and provided as asynchronous online lectures [6, 8, 24, 26, 31, 48, 52, 53] or screencasts [14]. 19 (28.4%) of the included sources of evidence analyzed virtual learning environments that were either self-conducted on the learning management system [25, 49–51] of the university or made available by external providers as Ecofisio [35, 72], Rheumatoid Arthritis for Physiotherapists e-Learning Platform [61, 80], King’s eLearning and Teaching Service [14], Physiotherapy eSkills Training Platform [63], C1D1 [24] or Nearpod [56]. One study reported using a massive open online course [70], one used “cursos on-line”, a multimedia online tool [76] and the remaining did not provide explicit information about the frame of the virtual learning environment used in their studies. The content was mainly delivered as videos (e.g. for the presentation of skills [12, 22, 24, 25, 32, 35, 39, 41, 43, 48, 49, 51–56, 59, 63, 69, 70, 72, 74–76, 78, 79, 82]), via written case scenarios or other written theoretical information [22, 25, 38, 41, 51, 55, 61, 63, 66, 72, 75, 76, 82], images presented two-dimensional or three-dimensional, sometimes interactive [22, 30, 35, 37, 38, 55, 56, 71, 72, 76]. Podcasts were utilized to provide information either in audio format [24, 50–53, 55] or as video-podcast [45]. In 4 (6%) sources of evidence, the content was delivered by power-point slides for asynchronous remote use [14, 26, 56, 82] and in 3 (4.5%) sources of evidence, the syllabus was uploaded [34, 49, 50]. Blog posts were used for the distribution of information in 2 (3%) sources of evidence [65, 82]. Vision-based augmented reality was only used in one study to facilitate the learning of anatomical structures (46). Additional information was provided via links to external websites (e.g. links to websites of international and national scientific societies) for further references [25, 49, 50, 53, 56].

Besides the beforementioned digitally enhanced educational methods, there were 24 (35.8%) sources of evidence using a gamification-based approach to facilitate learning motivation in students [14, 25, 32, 36, 39, 41, 43, 44, 49–51, 56, 58, 64, 66, 68, 70, 77, 82, 84]. For content delivery, 3 (4.5%) sources of evidence used virtual reality simulations and 2 had access to high fidelity mannequins. In one of the virtual reality scenarios, the students were asked to manage a patient as a physical therapist and received access to the information needed to diagnose this patient [66]. Ulrich et al. (2023) showed a 360° video of correct supine positioning via a virtual reality head mount display as a non-immersive way to use the device [77]. In the third study, the students tested virtual reality games to be used with patients in neurological rehabilitation as part of the “physiotherapy in neurological diseases” course [64]. The high fidelity mannequins were either used in a simulation-based educational scenarios or in an escape room scenario [39, 44]. Students had to build a physiotherapy diagnosis based on the symptomatology of the advanced simulation mannequin [39] or targeted other clinical domains [44].

Escape rooms that incorporated puzzle solving and using clues to complete activities and thus escape from the room within a certain amount of time, were analyzed by 2 (3%) different sources of evidence [39, 84]. Students had to decipher hidden messages, open locks and correctly carry out a prior learned treatment protocol in order to escape. Content delivery methods are displayed in table III (supplementary material).

## Briefing and debriefing

Discussion boards and online forums were used for student interaction, briefing and debriefing of learning sessions often by using the learning management system platform of the university or twitter (*n* = 9; [25, 32, 41–43, 47, 49, 50, 60]). Furthermore, social media groups (facebook [70, 81], online platforms [54, 56, 82] and e-mail [6] were used for communicational purpose.

### Knowledge gain and improvement in skill performance

Fortyseven (70.1%) sources of evidence analyzed the effect of digital (enhanced) education on knowledge gain and skill performance. Of these, 6 (9%) reported a significant positive effect either on theoretical knowledge [34, 61] or skill performance over time [24, 34, 50, 55, 65]. Additionally, 28 (41.8%) sources of evidence compared the effect on performance or knowledge of digitally (enhanced) education with traditional or less invasive (i.e. pre-recorded video tutoring vs. student produced self-video [74]) digitally enhanced teaching methods and found significant differences. Of these, 18 (26.9%) reported significant increases of theoretical knowledge or clinical skill performance in favor of the group receiving the digital learning method under study in at least one of their outcome measurements [6, 14, 31, 35–38, 41, 43, 46, 49, 51, 69, 71, 72, 74, 76, 78]), while 9 (13.4%) of the remaining sources of evidence found no significant differences between the experimental and control groups [12, 39, 54, 56, 59, 67, 75, 77] and one found a difference in favor of the control group that received a clinical case presented in text form instead of via immersive virtual reality (*p* = 0.023; [66]). The remaining sources of evidence did neither report the significance nor effect sizes of their findings.

The most used gamification approach for knowledge testing was quizzes, that were provided in different formats (e.g. drag and drop, Kahoot!, multiple choice; [14, 25, 32, 36, 39, 41, 43, 49–51, 56, 58, 68, 70, 82, 84]). One additional study (1.5%) examined “Physiotherapy Party” as a content delivery and knowledge testing method, that included mimicry, questions, forbidden words and drawings [84].

### Students’ satisfaction, perception and experiences

The overall student satisfaction was measured in 18 (26.9%) sources of evidence with a positive outcome in 16 (23.9%) sources of evidence, defined as a satisfaction score exceeding the midpoint of the measurement scale (i.e., > 50% of the maximum possible score) [6, 22, 30, 35, 36, 38, 40, 43, 52, 55, 56, 64, 70, 72, 75, 78]. In these sources of evidence, outcomes greater than 50% of the maximum possible score were considered indicative of a positive response to the digital learning method. This criterion was applied across various measurement tools, including Likert scales and numeric rating systems, ensuring consistency in the interpretation of satisfaction levels [6, 22, 30, 35, 36, 38, 40, 43, 52, 55, 56, 64, 70, 72, 75, 78]. One study reported a significantly better student satisfaction in the traditional learning group compared to two different styles of digital learning (video and 360° video) [77]. Etoom et al. (2023) analyzed distance learning in a cross-sectional online survey with a comparable low satisfaction score of 17.14/40 [23].

The improvement of the learning experience has been measured in 8 (11.9%) of the included sources of evidence with ambiguous results. In half of the sources of evidence, students rated that their learning improved with the use of digital methods [30, 60, 68, 78]. As before, the students in the study by Ulrich et al. (2021) favored the traditional teaching method with a significant difference [77]. In Nicklen et al. (2016), only 21% of the students experienced an improvement of the learning experience with the use of a remote-online case based scenario [62]. There was a difference in cohorts analyzed in Luedtke et al. (2022), with the second year cohort rating their learning experience as improved (6.14/10) and the first and third year cohort did not experience a strong improvement (3.04/10 and 4.41/10, respectively) [12].

Furthermore, 15 (22.4%) sources of evidence rated the delivery method of the learning content as appropriate (> 50% of the individual score, defined as above for the students satisfaction) [22, 25, 30, 34, 35, 44, 49, 50, 55, 56, 59, 60, 72, 83, 84]. Web-based distance education or remote-online case-based learning was rated as not suitable for content delivery in only two sources of evidence [33, 62].

Some advantages and disadvantages of the change from traditional face-to-face teaching to digitally enhanced teaching were reported, with an improvement of dedication and motivation analyzed by 16 (23.9%) sources of evidence [14, 22, 34, 36, 38, 43, 49–51, 55, 58, 59, 64, 77, 78, 84]. Only Ulrich et al. (2021) reported a significant difference to the traditional teaching method in favor of the face-to-face teaching [77]. The detailed information about the quantitative results for students’ satisfaction, perception and experience can be obtained in Table II (supplementary material).

Five (7.5%) sources of evidence reported that teaching staff received strong support in adapting their face-to-face teaching content into online formats, a factor that was particularly crucial during the SARS-CoV-2 pandemic.

**Fig. 3:**
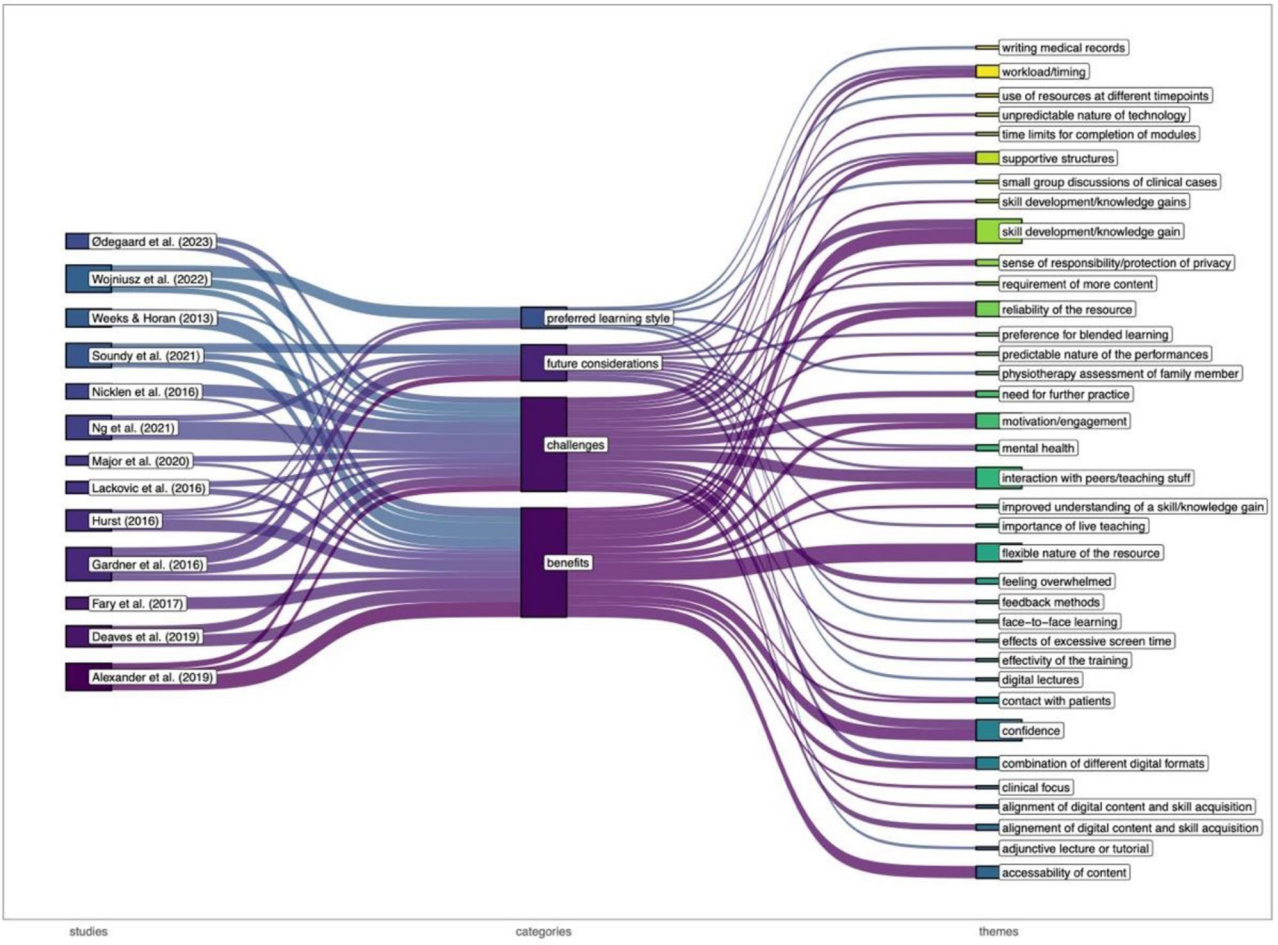
Sankey diagram of the summarized categories and themes identified by the qualitative sources of evidence

Adding on the beforementioned quantitative findings, 13 (19.4%) sources of evidence followed a qualitative research design to gain an insight into reasons for students’ satisfaction, attitude and perception with digital education. An essential finding was a dissatisfaction with the rapid changes in teaching modalities due to the pandemic reported by Ng et al. (2021; [8]). The key themes of the qualitative sources of evidence were synthesized and categorized into overarching categories. Common subcategories were identified and extracted. The result of the narrative synthesis is displayed in Fig. 3.

## Subjects/courses

The digital learning and teaching method was implemented in either theoretical courses, practical courses or skill trainings, with musculoskeletal courses being the most. Practical courses focused on providing knowledge related to disease-specific physiotherapy, while skill training sessions were designed to develop and refine practical skills through hands-on practice, emphasizing technique and competency in performing specific tasks. The course distribution is shown in Table 2.

**Table 2:** Courses in that digitally enhanced teaching was implemented

Theoretical courses	• professional ethics [34] • professional and multidisciplinary team issues [60] • evidence based practice [25, 47, 66] • viva preparation [79] • abdomen and pelvis anatomy [69] • musculoskeletal anatomy [32, 37, 42, 46, 71] • neuroscience [41]
Practical courses	• respiratory pathologies [44, 67, 77] • cardiac rehabilitation [39, 44, 49, 50] • rheumatoid arthritis [61, 86] • lymphatic issues [48] • ICU/first aid [83, 85] • pediatrics [82] • neurological rehabilitation [44, 65, 71] • obgyn [62] • musculoskeletal therapy [6, 24, 44, 51, 53, 56, 57, 73, 75, 76, 80, 85] • chronic condition therapy [31, 80]
Skill training	• manual therapy [12, 63, 72] • fit-ball [82] • ultrasound skills [35, 38, 70] • clinical practice skills (not further defined) [8, 22, 45, 52, 54, 57, 64, 78]

## Discussion

The large number of sources of evidence addressing digitally enhanced education in physiotherapy included in this review, along with the significant increase of related publications (e.g., studies on digital education in health professional programs) highlights the increasing interest and relevance of digital technologies in this field.

Looking at the results for knowledge and skill gain, most of the sources of evidence included in this review showed either a positive effect of the digital enhanced education or no significant difference to a control condition. Most of the sources of evidence reporting the overall satisfaction of the students had a positive outcome as well, but only with a small number indicating that the change of educational methods led to a positive impact on the learning experience. However, this review presents highly heterogeneous results with a high variety of digital educational methods and no clear consensus for or against individual methods can be given. Notably, digitally enhanced teaching seems most accepted and effective in the theoretical and basic knowledge transfer, particularly as preparation for skill acquisition training. The results of this review complement the results of existing publications on this topic by adding sources of evidence conducted during the Sars-COVID-19 pandemic and focusing on the students’ satisfaction next to the knowledge gain [17–19].

### Impact of COVID-19, challenges and limitations of digital learning

The Sars-COVID-19 pandemic was pointed out as the major facilitator for implementing digital education approaches with varied perceptions of distance learning in many of the here included sources of evidence [5, 9, 13, 23]. Due to the pandemic restrictions, face-to-face learning or even blended learning strategies were not possible. Solely digital content was created to substitute for the traditional teaching methods. While both synchronous and asynchronous online teaching methods offer certain advantages, intensive consideration is needed regarding the effective delivery of learning content in digital formats and appropriate pedagogical strategies to increase learning engagement [86]. The results of this review indicate that digital learning formats may also limit direct exchange of knowledge, skills and experiences between students and teachers, which could affect confidence in the knowledge and skills acquired [21, 59].

Another considerable factor to improve digital learning experiences and a lesson learned from the rapid change in teaching modalities caused by the pandemic is, that universities must provide adequate support for teaching staff in economical, organizational and technological aspects to ensure high educational standards when adding digitally enhanced teaching formats to the curriculum [6]. This includes the provision of technical support, training in distance learning and the development of effective workload management skills for the teaching staff [6, 22, 33, 38, 60, 72, 87].

Despite the rapid adoption to the teaching possibilities during the pandemic, some respondents in the included sources of evidence expressed dissatisfaction with digital learning compared to the traditional face-to-face teaching methods experienced prior to the pandemic [8, 12, 23]. This dissatisfaction led to recommendations for curriculum design changes, emphasizing a blended learning approach that integrates online resources with face-to-face practical skill development [8].

### Blended learning: the preferred method

Adding to recent research, the results of this review support the use of blended learning approaches in physiotherapy education for reasons of knowledge gain, improvement of skill performance and students satisfaction [19]. The combination of traditional and technology-based teaching approaches also seems to be successful in the improvement of the clinical performance and increase of students’ confidence in their clinical practice. Notably, Evans et al. concluded in their systematic review (2019 [88]), that there is no clear evidence for better academic outcomes in health profession students after changing to flipped classroom as teaching compared to traditional teaching. However, they observed that flipped classroom teaching produces academic outcomes comparable to those of traditional methods [88]. A preference for blended learning over purely online learning was observable in the results of this review, which could be attributed to its ability to better accommodate students’ preferences through increased flexibility (e.g., offering asynchronous or online content that allows for flexible study hours and locations). Additionally, blended learning may more effectively promote active learning by combining the benefits of face-to-face interactions with engaging, interactive online components, as described by Ødegaard et al. (2021; [19]).

Student-centered or active remote learning does allow students to learn at their own pace increasing learning engagement [89]. But special interest has to be on feedback opportunities that are reported as often missing in the digital learning offers [8, 24]. Additionally, digital methods often offer individually adaptable learning paths supporting the development of specialized knowledge [6, 24, 44, 53, 56, 58, 75, 84]. Our review found that students especially appreciated the versatility and audio-visual nature of student-self-videos as tool in preparation for practical examinations.

### Enhancing student satisfaction

It is important to consider students’ expectations, preferences and personal relevance (meaningfulness of the content for intrinsic motivation) in educational planning [21]. The increase of knowledge is positively correlated with the satisfaction of the students, hence, improvements of average exam results are estimated by consideration of the students satisfaction while learning [14].

To increase students’ satisfaction, the support provided by the lecturer plays a decisive role, especially in online or distance learning [23]. Interestingly, students, that are experienced in distance education, are often more satisfied in follow up courses. This could be due to appropriate teaching methods or to predictable expectations [23]. Additionally, the results of this review indicate that gamification is one learning modality that increases the satisfaction of students.

Gamified e-learning concepts can optimize training of physiotherapy skills [14, 25, 32, 36, 39, 41, 43, 49–51, 56, 58, 66, 68, 70, 77]. In particular, virtual reality technologies represent an effective resource for knowledge transfer and can play a fundamental supporting role, underlining the benefit of innovative teaching technologies in the development of clinical skills in training sessions and in clinical placements [8, 22, 45, 53, 57, 63, 77]. On another note, students who use quizzes (e.g. Kahoot!) and reward cards as learning tools demonstrate better academic results on knowledge gain with the reinforcing component of being tested compared to those without reinforcement [14, 25, 32, 36, 39, 41, 43, 49–51, 56, 58, 68, 70, 82, 84]. Hence, this methodology can be an effective tool to promote student engagement and support content acquisition as well [36].

Immersive (360°) videos are characterized by their cost-effectiveness and have a positive influence on the user’s emotional reaction to the learning environment and therefore might be considered as content delivering method. They represent a viable alternative to virtual reality and conventional videos. However, the results by Ulrich et al. (2021; [77]) indicate that traditional teaching is either equivalent or even more effective than using of 360° videos in most aspects.

Students who were offered an escape room learning scenario, have gained extensive knowledge on the provided subject. Additionally, the qualitative comments of escape rooms as teaching method were fairly positive [39]. The methodology of case studies and role plays were rated as the most effective techniques in knowledge transfer by students [34].

The application of gamification (as described before: online quizzes, immersive videos, escape rooms) proves to be extremely effective in enhancing student satisfaction in other health professions as well [90]. Innovative learning methods that combine curiosity and intrinsic motivation with the transfer of knowledge seems to arouse particular interest among learners [39, 44, 56, 64, 84]. Furthermore, there seems to be potential in focusing on cognitive stimuli through playful elements and thus sustainably increase learning engagement and students satisfaction [25, 39, 43, 44, 56, 58, 64, 84]. The role of motivation in gamification remains a topic of debate. From a theoretical standpoint, motivation in gamified contexts is often considered to be more extrinsic than intrinsic. The reward based extrinsic motivation yields solely short-term engagement [91].

### Future directions

The findings of this review support recommendations to adopt a blended learning approach in future programs. Additionally, implementing online formats could enhance access to education for future physiotherapists, given the opportunity to partially learn remotely, hence, more flexible. A transition to e-learning could also be considered specifically for additional parts of physiotherapy undergraduate programs to thoroughly facilitate access to tertiary education [6].

There is a clear need for further research to determine the best possible design of blended learning courses in physiotherapy education. In addition, it is essential to develop strategies to improve the effectiveness of distance learning. Further research is particularly necessary in clinical placements to improve technology-enhanced learning and to address the decline in perceived self-efficacy among students [6, 12, 21, 54, 62, 73, 79, 92].

### Limitations

The comparability of the evaluated sources of evidence is limited due to heterogeneity in design, digitally enhanced teaching and learning methods used, and in the recording of results.

Due to the extensive number of sources of evidence included, the character of the present review is mainly descriptive.

Some of the sources of evidence integrated online or hybrid teaching methods used in the context of the COVID-19 pandemic, which implies a rapid adaptation from face-to-face teaching to online teaching alone. This may differ significantly from online methods that have been developed, tested and optimized over longer periods of time.

The temporary switch to a fully digital learning approach in physiotherapy education appears to intensify the extent of negative emotions. In addition to the rapid adaptation of learning modalities and existing uncertainties within a study program, many students reported changes in other areas of life, such as job loss or loss of social support systems. Such changes presumably had an impact on students’ psychosocial well-being and on their perceptions while learning.

## Conclusion

The present review provides an overview about different teaching strategies used for distance education or digital enhanced education in physiotherapy degrees.

Both quantitative and qualitative sources of evidence included in this review show that the knowledge gain, learning experience and satisfaction of physiotherapy students could be increased by digitally enhanced educational opportunities. Gamified learning strategies facilitated the engagement of students best in this review, although further research is required to validate its effectivity on knowledge gain and further outcomes.

## Electronic supplementary material

Below is the link to the electronic supplementary material.

Supplementary Material 1

Supplementary Material 2

Supplementary Material 3

## Data Availability

No datasets were generated or analysed during the current study.

## Abbreviations

APP: Assessment of Physiotherapy Practice
ASES: Academic self-efficacy scale
AQPEPT: Attitudes Questionnaire towards Professional Ethics in Physiotherapy
BHT: Bronchial hygiene techniques
BL: Blended learning
BLG: Blended learning group
C1D1: See one, do one
CBL: Case-based learning
CG: Control group
CI: Clinical instructor
CP: Cardiopulmonary
DE: Distance education
DELES: Distance Education Learning Environments Survey
DL: Distance learning
DREEM: Dundee Ready Educational Environment Measure
EBP: Evidence based practice
EBPQ2: Evidence-Based Practice Questionnaire
EE: Educational environment
EER: Educational Escape Rooms
EG: Experimental group
ES: Exercise Science
EP: Exercise Physiology
FCM: Flipped classroom model
FG: Face-to-face learning group
LMS: Learning management system
LORI: Learning Object Review Instrument
MCQ: Multiple Choice Questionnaire
MEAH: Model of emotions, adaptation and hope
MLE: Maximum likelihood estimation
MOOC: Massive open online course
NMS: Neuromusculoskeletal
OL: Online learning
OLSES: Online learning self-efficacy scale
OSCE: Objective structured clinical examinatron
PTIP: Physiotherapy
PTIP: Physiotherapy teaching innovation project
PVT: Pre-recorded video tutoring
RA: Rheumatoid arthritis
RAP-eL: Rheumatoid Arthritis for Physiotherapists e-Learning
RO-CBL: Remoteonline case based learning
S4BE: Students for best Evidence
SBE: Simulation based education
SFS: Student Feedback on Subjects
SMS: Self-management system
SNS: Social networking sites
SSV: Student produced self-video
TEL: Technology enhanced learning
VR: Virtual reality
