# Supplementary material for: Overview of styles, content, learning effects and attitudes of students towards digitally enhanced physiotherapy education – a scoping review
Source: BMC Med Educ. 2025 Feb 4;25:176. doi: 10.1186/s12909-025-06750-6 (PMC11792568; doi:10.1186/s12909-025-06750-6)
Supplement: Supplementary file 1 — Supplementary Material 1 [file 12909_2025_6750_MOESM1_ESM.docx]

| title | authors | study design | population | number of participants (n) | inclusion criteria | exclusion criteria | form of education | detailed form of education | intervention | comparison | outcome | results | positive impact of digital education |
| --- | --- | --- | --- | --- | --- | --- | --- | --- | --- | --- | --- | --- | --- |
| A blended-learning programme regarding professional ethics in physiotherapy students | Aguilar-Rodriguez et al. (2019) | prospective simple-blind clinical trial | third year degree physiotherapy students | 129 | studying the physiotherapy degree at the University of Valencia | prior training on ethics subject | hybrid | blended-learning programme (based on professional ethics and related to clinical practices) | Both groups: clinical practices in hospitals and clinical centres during 8 months. Experimental group: followed an 8-month blended-learning programme based on professional ethics, including two phases: (1) online syllabus and online activities (4 months); and (2) face-to-face group sessions (4 months). | Case study method; no online learning or online activities: students had to create and to perform a public exposition of a real clinical case. Cases had to be related to clinical practices in order to recognize ethical situations in the work environment. | attitude towards learning professional ethics (baseline, 8 months): Attitudes Questionnaire towards Professional Ethics in Physiotherapy (AQPEPT); knowledge (baseline, 4 months, 8 months): perceptions about knowledge regarding professional ethics in physiotherapy; opinion towards learning professional ethics (4 months, 8 months): questionnaire comprising eight dichotomous questions and one open question for syllabus + questionnaire comprising five items and one open question for face-to-face) | Attitude: significant differences were found for the EG group, whereas these differences were not observed in the CG; favourable responses of EG increased a 30%, while indifferent and unfavourable responses decreased a 13% and a 17%. Favourable responses of CG remained unchanged (49%), whereas indifferent responses showed an increase of 3% and unfavourable responses showed a decrease of 3%. Knowledge: no significant differences within group in CG. EG group showed a statistically significant increase in knowledge towards professional ethics at the end of the professional than at baseline or at 4 months. Opinion: Students considered an appropriate extension of the syllabus (100%), appropriate timing of the programme (94%), adequate specificity of the syllabus (91%), adequate comprehension of the syllabus (86%), usefulness of online activities (90%), usefulness of associating clinical practices and professional ethics syllabus (84%), appropriate online method (83%) and 88% would recommend the syllabus topics. Regarding the methodology developed during face-to-face learning, students highlighted the importance of clinical practices when learning professional ethics. | yes |
| Technology-enhanced learning in physiotherapy education: Student satisfaction and knowledge acquisition of entry-level students in the United Kingdom | Alexander et al. (2019) | mixed methods (focus group interviews + clinical trial) | first year physiotherapy students (bachelor and masters degree) | 79 | NI | NI | hybrid | King’s E-Learning and Teaching Service (virtual learning environment), blended learning approach | A variety of technology-enhanced learning (TEL) ressources were created and made available for the students: Power point, quizzes, screencasts etc. | previous study cohort (without access to the TEL ressources) | validity and efficiency of evaluation of online learning resources: Learning Object Review Instrument (LORI) (adapted) / Learning analytics data: Moodle Learning Analytics Tool / knowledge acquisition: practical and written examination results from the 2015/2016 study group cohorts were compared with the same examination results of the previous 2014/2015 cohorts. Themes about students satisfaction and perception of usefulness for knowledge acquisition: focus group interviews (3 groups) | Forty-three (55.1%) students responded to the survey, with 42 students completing all questions. 97.6% agreed or strongly agreed that they were satisfied with the resources, whilst 76.7% agreed or strongly agreed that the resources were engaging and interesting; 97.6% of respondents agreed or strongly agreed that the resources helped them with their practical and written exams, and 100% agreed or strongly agreed that the resources were useful in aiding knowledge of exercise medicine. Focus groups: five higher order themes: (1) Content quality, (2) Interaction and accessibility, (3) Learning goal alignment, (4) Satisfaction with resources and (5) Suggestions for the future / Learning analytics: module was accessed 10 548 times, most accessed resources were the strength training quiz, module news, updates forum and module handbook. There were noticeable peaks in usage in the weeks preceding written and practical exams. Exam results: statistically significant improvement in the mean mark for the practical and written exams as well as overall module grade, overall mean module grade improved from 67.6% to 75.1%, the mean practical examination mark increased from 72.9% to 81.8% and the mean written examination mark increased from 67.62% to 70.8% | yes |
| Students 4 Best Evidence as a digital Problem-Based Learning method to improve Evidence-Based Practice competencies in undergraduate physiotherapy students: an observational study | Arienti et al. (2020) | observational study | first year physiotherapy students (bachelor degree) | 121 | NI | NI | digital | digital problem-based learning with Students for best Evidence (S4BE) blog | Evidence based practice (EBP) laboratory: 24 educational training hours using S4BE blog, carried out over a 5-month period, with a total of six*4h sessions. The first five sessions were completed during the first 3 months, and the last session was completed after 2 months of clinical training within a hospital. EBP curricula model: set of EBP core competencies (68 competencies, grouped in five domains: introductory, ask, acquire, appraise and interpret, apply and evaluate). The students were divided into three or four different working groups composed of six or seven students and, during each session, they were given a specific ‘scientific problem’ to solve. | NA | EBP competencies (baseline and end of laboratory): Evidence-Based Practice Questionnaire (EBPQ2) | drop-out rate of 15%. EBP competencies: significant improvement in all domains (p<0.001), except in the sympathy domain, where the percentage score decreased from 71% to 60%. The best improvements were reached in terminology (54% to 65%) and in practice (41% to 55%) domains. | yes |
| A blended learning approach to palpation and ultrasound imaging skills through supplementation of traditional classroom teaching with an e-learning package | Arroyo-Morales et al. (2012) | randomized controlled trial | second year physiotherapy students | 46 | NI | previous education/training in musculoskeletal ultrasound imaging | hybrid | blended e-learning | both groups: 2x2h training session, 1. session: theoretical setting, 2. session: analysing 5 structures of the knee, palpation, ultrasound examination / experimental group: access to the ECOFISIO website (for 3 weeks); content: videos of the palpation procedure, image showing correct placement of the ultrasound probe, diagram of the ultrasound scan, ultrasound slice, anatomical section; self-assessment procedure on the skills acquired | books and texts on the topic but not to the website (for 3 weeks) | Palpation skills + ultrasound imaging: objective structured clinical evaluation (OSCE); Knowledge of ultrasound physics, ultrasound technology, clinical applications, anatomy: Multiple Choice Questionnaire: (20 questions); Time taken by the student to generate a reliable ultrasound image and to localize a specific knee structure by palpation; Evaluation (5-point Likert scale): quality of the educational intervention, competence of the teacher, acquisition of knowledge+skills, interest in participating in the study of another anatomic region, satisfaction with the ECOFISIO website, preferation to be in another study group | 61.6% of participants used the web page for >1h/day during the study period / theoreticial knowledge acquisition: no significant difference between control and experimental groups (mean scores of 7.42 and 7.23 / maximum of 10 points) / OSCE + time: experimental group needed less time (36.60 vs. 54.10s) to palpate the musculoskeletal structure in question and scored higher for the individual item positioning of the patient for palpation. Experimental group students took longer (99.09 vs. 77.60 s) to acquire the ultrasound image, but their ability to correctly position and manage the probe was superior. Global scores for the OSCE components on knee palpation and ultrasound examinations were significantly higher in the experimental group palpation: p=0.041; ultrasound: p<0.001); some OSCE items on palpation skills were not significantly higher in the intervention group (only "positioning of patient": p=0.045). Scores for some OSCE items on ultrasound skills did not significantly differ between groups (only "orientation of ultrasound probe" p<0.001 + "handling of ultrasound probe": p<0.001). Participants evaluation: both groups were interested in learning another anatomical region (Likert score >4.2) although the interest of the control group was higher; neither group would have preferred to be in the other learning program (Likert score <1.7); a high level of satisfaction with the characteristics of the website (>4.1 in Likert scale). | yes |
| Not letting a good crisis go to waste – learnings from a fully online professional placement | Barradell et al. (2023) | mixed methods approach | Master of Physiotherapy students | 16 | enrolled in the unit in 2020 | NI | digital | online unit (replaced one of the program’s professional clinical practice placements) | online unit was offered three times across 2020 (i.e. some students were on placement while others were learning online). Content: simulated case notes, including videos, photos and scans, were utilized in almost all activities. There were three assessments: 1) completion of, and participation in, weekly activities contributed to a continuous assessment for learning (individual); 2) a series of case-based tasks over a week that contributed to formative assessment (individual); and 3) a group discussion board related to prioritization of a service caseload that was also a formative assessment. | NA | student experience: survey (open-ended + closed questions); Practice thresholds + competence: Assessment of Physiotherapy Practice (APP) | Experience: - closed questions: very high levels of satisfaction across the surveyed areas with a mean score of 8.45 of 10 points for all questions summarized. - open-ended questions: majority of participants were satisfied with the unit and indicated that the unit helped them to learn valuable skills and that they were able to apply their learning to future contexts. Specific cases and modules based around healthcare settings rather than streams appeared to help students to consolidate their past learning. Students seemed to be appreciative of the response made to keep them progressing through the course and clinical placement suites. Students generally found feedback from educators to be helpful and particularly enjoyed the synchronous classes, which provided an opportunity to formally connect with peers, staff and content. Some students found the workload for the unit high. Others appreciated the accountability that came with regular deadlines; key advice to other students was focused on time management. Suggested areas of improvement related to timing of release of case information and quantity of feedback. Small numbers of students were also ambivalent about the use of online media and tools including discussion boards and being part of a learning community. | yes |
| An Investigation of Academicians and Students' Perception and Attitudes for Distance Physiotherapy and Rehabilitation Education in COVID-19 Pandemic | Bayrak et al. (2021) | observational study | academicians (left out in the rest of this table) and undergraduate physiotherapy students | 381 students | NI | NI | digital | distance education (DE) | no further information about the intervention | NA | students perceptions: online survey; students’ attitudes towards web-based instruction: Web-Based Instruction Attitude Scale | 160 (42%) of the students had previously participated in a DE program; Web-Based Instruction Attitude Scale: total score was 72.11±20.29 (range=26-130); - efficiency of Web-Based Instruction: 49.01±16.16 (range=17-85); - to Resist Web-Based Instruction: 23.10±8.09 (range=9-45); online survey: DE was an alternative solution to the education problem (56.20%) in the period of COVID-19. Students did not feel that they have belonged to the university they were enrolled in (50.40%). DE was not as effective as face-to-face education (58.80%). | no |
| Immersive virtual patient simulation compared with traditional education for clinical reasoning: a pilot randomised controlled study | Bonnin et al. (2023) | pilot randomised controlled study | 3rd year physiotherapy students | 48 (23 in experimental group, 25 in control group | NI | (1) non-corrected visual disorder, (2) contraindication for using VR (epiilepsy, risk of convulsion, pacemaker, implantable medical devices), (3) taking drugs altering memory or attentional capabilities | digital | clinical case via immersive virtual reality | course: respiratory pathologies; 45min to individually resolve the clinical case (physiotherapy asessement and diagnosis) on double-sided copies. Instructions were given on a seperate sheet of paper and the students were asked to manage a patient as a physical therapist. Concerning the diagnosis, the students were told that notions of physiopathology were expected (e.g. diaphragmatic insufficiency due to thoracic distension). They had to name therapeutic means without detailing them (e.g. exercise training). In VR the students were placed in a virtual consulting room and could consult the clinician's computer, the patient or the examining table. Students could make use of: (1) consult general information and complementary examinations (e.g. functional complementary tests, six-minute-walking-test, stress test, pulmonary scintigraphy) by clicking on the clinician's computer (2) to see the patient describe the difficulties encountered in carrying out activities of daily living and leisure activities by 360° video (3) to observe patient auscultation by 360° video | clinical case via text (same information as in the VR group, but in textform | clinical reasoning performance: exam score; Perceptions about clinical cases, experience in VR: survey (dichotom, 4-point-Likert-Scale); Sense of presence: Slater-Usoh-Steed-Questionnaire (SUSQ) | clinical reasoning performance: EG had significant lower total score of 20 points than the CG (p=0.023, respectively 9 [8:10.5] vs 11 [9:14]. There was a significant difference between groups regarding patient assessment (10 points) (p=0.0010, EG: 3.5 [3:4.5] vs. CG: 5 [4:5.5]) but none for diagnosis (10 points) (p=0.635). Concerning assessment, only patient history showed significant lower scores for the EG compared to the CG (p=0.007, EG: 2 [1.5:3.5] vs. CG: 2.5 [2:3.5]). Perceptions about clinical cases: tolerance of VR was significantly related to performance results (p=0.04). | no |
| Student physiotherapists perceptions of online curriculum delivery during the COVID-19 pandemic | Chesterton et al. (2022) | cross-sectional exploratory survey; mixed method approach | students of undergraduate or postgraduate degree in physiotherapy | 236 | NI | NI | digital and hybrid | online learning and hybrid learning models | no detailed information about the intervention | NA | students' attitudes, experience and satisfaction with the transition to online learning: Online Questionnaire (dichotomous, multiple choice or Likert scale; open ended) | 51% (n=119, 95% CI 44 to 57) were ‘apprehensive about the change’, 33% (78, 27 to 39) ‘could see the benefits/opportunities of using technology’ and 10% (24, 7 to 15) were ‘not worried/concerned’ with the transition. 159 respondents (67%) either ‘strongly disagreed’ (32%, 76, 27 to 39) or disagreed (35%, 83, 30 to 42) that they were equally motivated to learn using an online platform compared to a face-to-face traditional approach. The lack of daily structure, distractions during sessions, lack of cohort interaction and ability to practice hands-on skills were reasons cited. The opportunities to engage in online classes were not considered the same as face-to-face (63%, n=149, 95% CI 57 to 69). 55% of respondents felt academic staff and lecturers had the necessary skills to deliver effective online content. Students identified the often-shorter sessions were time efficient. The convenience of speaking to tutors and the safety of the home environment during the pandemic were also considered advantages of an online learning delivery. 79% of respondents felt online learning had a negative impact on their understanding of the subject area. The primary reason for this was a lack of confidence in applying clinical and practice skills (163, 88%, 82 to 92), followed by connectivity issues experience during classes (71, 38%, 31 to 45) and the pace of session delivery (70, 37%, 31 to 45). For the 50 respondents who felt online learning had not impacted negatively on their learning experience, the use of technology (33, 66%, 52 to 78) and availability of recorded sessions (28, 56%, 42 to 69) had allowed for better subject understanding. Respondents in the main preferred a mixture of synchronous and asynchronous online delivery (60%, 142, 54 to 66). | no |
| Study Based on Gamification of Tests through Kahoot!™ and Reward Game Cards as an Innovative Tool in Physiotherapy Students: A Preliminary Study | Cortés-Pérez et al. (2023) | experimental design | 1st to 4th year physiotherapy students | 313 | (1) students enrolled in the Degree of Physiotherapy from University of Jaén; (2) currently taking one of the four subjects participating in this physiotherapy teaching innovation project (PTIP); and (3) participating voluntarily and being able to report on the various dimensions of the PTIP in the subject that they are enrolled in. | NI | hybrid | gamification (Kahoot! quizzes) | Quizzes are presented in game mode, in which the student’s mobile device or laptop becomes a remote control with four buttons. Each question in the questionnaire presents four response options. It is possible to select an answer to the question that the teacher has launched from his computer, projector or electronic whiteboard. Playing cards are rewards for the winners of each Kahoot! session. The teacher responsible for each subject selected the theoretical content to be taught for 30h over a period of four months. 50% of the theoretical content of the subject was randomly selected to be reinforced with Kahoot! (8 questions with 4 answer alternatives) and game cards. The other half followed the traditional teaching model. After answering each question in Kahoot!, the teacher resolved any doubts about that question, as well as explained the reasons why one question was true and the other three were false, providing instant feedback to the students. | non reinforced theoretical content (no gamification/no reward) in the same subjects (+ same participants) | knowledge: multiple choice quiz (final written exam of each subject); Perception, innovation of the project, motivation, usability, general satisfaction with this PTIP: questionnaire | knowledge: significant difference was detected in the number of correct answers in the final test exam of each subject. Greatest difference was observed in the subjects of the first- and third-year course. More than 20% of correct answers in average terms (95% CI 17.61 to 26.86; p<0.001 for the first-year course and 95% CI 16.69 to 24.17; p < 0.001 for the third-year course) was obtained in the reinforced contents compared with non-reinforced contents. In the second- and fourth-year courses, the improvement in the percentage of correct answers was statistically significant but with less impact, almost 9% and 7%, respectively (95% CI 5.43 to 12.33; p<0.001 for the second-year course and 95% CI 3.85 to 9.38; p<0.001 for the fourth-year course); Satisfaction: in all the questions, the answers of “strongly agree” or “agree” resulted in the highest percentages among the participants of the different courses. More than 90% of the total participants considered that games with Kahoot! helped them to review concepts of the subject and clarify doubts; the students also think that gamification might be useful in other subjects. Approximately 90% of participants considered it motivating that Kahoot! quizzes were linked to reward cards and considered Kahoot! an innovative tool for teaching and learning. Kahoot! activities have motivated more than 65% of students to study every week (not only for the final exam). Almost all the participants were able to participate in the Kahoot! activities from home when the classes were online. | yes |
| Oncology E-Learning for Undergraduate. A Prospective Randomized Controlled Trial | da Costa Vieira et al. (2016) | prospective, controlled, randomized, and crossover study without blinding | undergraduate students of physiotherapy | 67 | (1) students enrolled in the 2nd, 3rd, and 4th year of the physiotherapy program, (2) ≥ 18 years, (3) weighted average grade ≥ 5.0 in previous academic years | hearing and/or visually impairing | digital | e-lecture | Students were randomized into two classes (class A or B) to attend lectures in which the same content was presented simultaneously, differing only in the teaching modality; one class attended a classroom lecture, while the other attended an e-lecture. Six modules (three modules/day), where each module corresponded to distinct content divided into three lectures, constituting a total of 18 subjects. The same content was simultaneously given to classes A and B. Class A: traditional classroom; at the content end, the students had 5 min for discussion with the teacher. Class B: e-learning classroom (storage material), using an individual computer and 5 min was given to study with the computer. The students could study the slides content, as there was no professor for discussion. First module: class A had a sequence of traditional/e-learning/traditional classroom and the class B had an e-learning/traditional/e-learning sequence. After each module (three lectures), the students had a minimum of 30 min to change to the other classroom. | traditional learning (normal classroom lecture) | knowledge: diagnostic and final or summative assessment (true, false, or do not know); Test (126 questions, prior to the beginning of the course + after the end of each module): objective assessment (summative evaluation); level of satisfaction with the different teaching methodologies and course content: open question | Knowledge: no difference between the classes was observed when comparing the respective students according to the teaching modality to which they were subjected (p=0.858). The grades were 9.30 (SD 0.83) for the classroom course and 7.87 (SD 1.23) for the e-learning course; Satisfaction: 52% of students reported that the topic added greatly to the knowledge. 57% (n = 38) of students considered e-learning superior or similar to the conventional classroom methodology. When asked if an extension course could be delivered remotely, 27% said yes, and 15% stated that the teaching modality did not influence learning. 21% said that a lack of communication with the teacher led to learning difficulties. | knowledge: no; satisfaction: yes |
| Improving physiotherapy students' anatomy learning experience and short-term knowledge retention—An observational study in Malta | Cuschieri & Narnaware (2023) | experimental design | first-year physiotherapy students | 22 | NI | NI | digital | Kahoot! game-based quiz | course: abdomen and pelvis anatomy study unit (2h/week); Kahoot! was used via a multiple-choice question setting, where students needed to choose the best answer out of four potential answers for each question. Each question was generated by the lecturer. The quiz was initiated 3 weeks after the start of the study unit for four successive sessions per quiz and was presented at the end of the lecture. Each student was invited to take part through a QR code. The quiz followed an open-book technique but the students could cast their answer only once in a timeframe of 20s. Once the time elapsed, the correct answer was shown before moving on to the next question. Students were given the floor to ask any questions or clarifications with regard to the question that had been just presented. | before initiation of Kahoot! quiz | Satisfaction: survey (5-Point-Likert-Scale); Short-term knowledge retention: answers given to the first 12 Kahoot! questions | short-term knowledge retention: positive short-term knowledge retention across time was noted with a significant increase in Kahoot! scores, as well as a significant increase in the frequencies of correctly answered responses. Four questions showed significant Kahoot! scores differences (p<0.05). Satisfaction: better experience following the initiation of the quizzes (χ2: 5.1, p-value: 0.02). All students agreed that the use of the interactive quiz improved their anatomy short-term knowledge retention. | yes |
| Students’ Perceptions of the Educational Value of Twitter: a Mixed-Methods Investigation | Deaves et al. (2019) | mixed methods investigation | final year undergraduate physiotherapy students | 33 for the baseline survey; focus groups: 7 novice user; 6 super user | NI | NI | digital | twitter feed postings / Twitter discussions | Twitter feed designed to supplement teaching and learning for one module: the module syllabus covered a variety of professional and multidisciplinary team issues within the wider context of physiotherapy practice. A variety of information and formats were tweeted based on the pedagogical principles of maximising student engagement. The tweets were in the public domain as this was deemed appropriate to model effective online professional behaviour. A short training session was provided to ensure that the cohort of students has access to Twitter and were orientated to its key features and functional abilities. | for the focus groups: novice users vs. super users (of social media) | social media usage and preferences: baseline survey; evaluation of students’ learning experience regarding the dedicated Twitter feed: follow-up survey; students’ experience: focus groups (six months after the implementation of the Twitter feed) | surveys: increase of 46% in the use of Twitter professionally at the end of the academic year. The students did agree with the 2 items: The Twitter feed provided me with useful information regarding the module + the Twitter account should be continued to support third year learning; focus groups: 3 major themes: issues related to digital literacy, educational experience and professional identity | yes |
| Pedagogical impact of integration of musculoskeletal anatomy blended learning on physiotherapy education | Delafontaine et al. (2023) | monocentric observational retrospective study | 2nd + 3rd year physiotherapy students | 92 (control group); 98 + 95 (experimental groups) = 285 | NI | NI | hybrid | blended learning | course: musculoskeletal anatomy; 2 experimental groups (cohort from 2018-2019 + 2019-2020): both groups had previously attended in-person gross anatomy courses and specific physiotherapy musculoskeletal anatomy blended learning. 32 musculoskeletal anatomy blended learning units were created. All blended learning units were broadcasted by the digital teaching platform and freely accessible for each student, had the same structure and duration (i.e., 30 min) and were pre-recorded with the same teacher’s voice. The blended learning units were composed of anatomy bullet text, 2D/3D musculoskeletal anatomy pictures (i.e., illustrations, diagrams and 3D models) and cadaveric musculoskeletal images. No e-video was included in the blended learning units. | traditional teaching methods (cohort from 2017-2018) | anatomy skills: final exam performance; evaluation of pedagogical value + interest of blended learning: survey | anatomy skills: improved success rate of the anatomy final written exam for both semesters studied. For the first semester, the mean score results to final exam of the control group, and of the experimental groups improved (Kruskal-Wallis = 74.06, df = 2, value of p<0.001). For the second semester, the mean score results to final exam of the control group, and of the experimental groups improved (Kruskal-Wallis = 173.6, df = 2, value of p<0.001). The mean score results to final exam of 2018–19 experimental group significantly decreased (Mann–Whitney = 6195.5, value of p<0.001) compared to the control group. The mean score results to final exam of 2019–20 experimental group significantly increased (Mann–Whitney = 163.0, value of p<0.001) compared to 2018–19 experimental groups. Pedagogical value: 74% (n = 66/89) of 2018–19 experimental group students and 80% (n = 58/72) of 2019–20 experimental group students said that blended learning could not replace in-person classroom lectures. 64% (n = 57/89) of 2018–19 experimental group students and 58% (n = 42/72) of 2019–20 experimental group students found that blended learning support is useful for learning musculoskeletal anatomy. interest of blended learning: 61% (n = 54/89) of 2018–19 experimental group students and 60% (n = 43/72) of 2019–20 experimental group students do not consider blended learning anatomy to be useful in preparing for the final written exam. | anatomy skills: yes; satisfaction: no |
| Distance learning in physiotherapy education during the COVID-19 pandemic: students' satisfaction, perceived quality, and potential predictors of satisfaction | Etoom et al. (2023) | cross-sectional qualitative online survey | all physiotherapy students in jordan (90% undergraduate) | 296 | experienced distance learning for at least one academic semester | NI | digital | distance learning (DL) | no further information about the intervention | NA | survey (multiple choice questions): preferred online tools, barriers and challenges in using DL, beliefs regarding the best and worst courses that can be delivered by DL; students perception of DL quality and satisfaction: Distance Education Learning Environments Survey (DELES) | 57% had previous experiences in DL. DELES-quality: good quality of DL (M=116.07, SD 22.5 of 170 points). DELES-satisfaction: not satisfied (M=17.14, SD=8.5 of 40 points). The predictors instructor support (t=5.37; p<0.001), personal relevance (t=2.44; p<0.015), previous experience in DL (t=3.241; p=0.001) and being a masters student (t=2.92; p=0.004) explained 30% of the variance in DELES-satisfaction. Best courses to be delivered by DL: university compulsory and elective courses (72.6%), general PT courses such as research methodology and ethics (30.4%), psychology (27.4%), anatomy and physiology (18.6%). Worst courses to be delivered by DL: PT speciality courses including practical or clinical training or clinical internship (54.5%), basic PT courses such as tests and measurements and therapeutic excercises (49.7%), neurological courses (48%), musculoskeletal courses (45.4%). Most preferred online tools for DL: YouTube (43.6%), Zoom Meeting (41.2%), Facebook live (29.7%), Moodle system (24.3%). Main challenges or barriers when using DL: internet connectivity (65.2%), distraction and lack of concentration (63.5%), feeling bored (49.3%), home environment (47%). | satisfaction: no; students rated the quality of DL content as good. |
| Assessing Implementation Readiness and Success of an e-Resource to Improve Prelicensure Physical Therapy Workforce Capacity to Manage Rheumatoid Arthritis | Fary et al. (2017) | qualitative: incorporating focus groups, and prospective, within-subject, cross-sectional, between-group phases | physiotherapy students | focus groups: n=23; prospective study: n=137 for short-term change; n=62 for long-term change; cross-sectional study: n=36 (historical cohort) + n= 62 | for particpation in the focus groups: being in the cohort that had access to Rheumatoid Arthritis for Physiotherapists e-Learning (RAP-eL) during 2014; prospective study: prelicensure students enrolled in a third-year unit of a 4-year physical therapy course; cross-sectional study: 2014 graduation-year cohort (historical control) with no access to RAP-eL + 2015 graduation-year cohort with access to RAP-eL | NI | digital | RAP-eL: the resource is supported by multimedia and practiceenabling tools that include 4 learning modules, 2 clinical case studies, interviews with consumers with rheumatoid arthritis (RA), evidence summaries, evidence tables, assessment tools, referral letters, and consumer resources. The modules are mapped to the natural history of RA | every student enrolled in the unit was required to complete the 4 modules and 2 case studies contained within RAP-eL, and the online assessment questionnaire, as a standard part of the unit’s curriculum and assessment requirements. | prospective study: short-term vs. long-term; cross-sectional study: historical cohort vs. actual cohort | acceptability, implementation readiness: focus groups; previous experience with online learning: online assessment questionaire | focus groups: the implementation of RAP-eL into the curriculum as a digital translation resource was determined to be acceptable to students. Five key characteristics of RAP-eL that supported this determination were identified: 1. in-depth insight into patient and treating-clinician perspectives 2. holistic approach to patient management 3. more clinically focused/practically based than typical lecture format 4. user-friendly interface and information 5. comprehensive, centralized digital e-learning resource; short-term change: all outcome measures demonstrated significant improvements after exposure to RAP-eL (p≤0.001); long-term retention: significant improvements from baseline were retained after 14 months for self-reported confidence in knowledge and skills to manage people with RA (p<0.001), prediagnosis vignette (p=0.02), and the clinical statement about atlantoaxial instability (p=0.048); the 2015 graduation-year cohort who had previously accessed RAP-eL demonstrated significantly better self-reported confidence in both knowledge and skills to manage RA, and responses to the early-stage RA vignette, than those of the 2014 graduation-year cohort, who had no previous access to RAP-eL (p=0.003, p=0.007, p=0.007). No other variables demonstrated significant differences between the cohorts. | yes |
| Validity and Reliability of Methods for Sonography Education in Physiotherapy: Onsite vs. Online | Fernández-Carnero et al. (2020) | exploratory observational prospective study | physiotherapy students | 136 | NI | NI | digital | online education of sonography | The onsite group received the programmed theoretical contents. The scheduled practice hours with sonographs were conducted by a musculoskeletal sonographer with 10 years’ experience. The online group used video recordings uploaded to a website with the same theoretical contents, scheduled practice hours (without the intervention of the musculoskeletal sonographer) as the onsite group. Practice time with sonographs devices (for both groups) was scheduled before the practical exams. The anatomical regions about which attendants were educated were upper limbs (shoulder, elbow, and wrist) and lower limbs (hip, knee, and ankle) for the two groups. | traditional education (onset) vs. online, with 2 subgroups: basic and average level | theoretical exam: Kahoot!; practical exam: using phantoms, five different measurements of two structures (filaments) included in the gel phantom (diameter of the filaments, distance between them and depth) | theoretical exam: In the Kahoot_basic scores, the model detected a positive, although not very high, difference between the Online 1 and Onsite 1 groups (0.6708, SE=0.2680) and a significant (t=2.502, p=0.0149) and negative and small difference between the Onsite 1 group and the total effect (−0.3354, SE=0.1340) and a significant difference (t=−2.502, p=0.0149) with a low effect size (R2adjusted=0.103). In the Kahoot_advanced scores, the model detected a positive difference between the Online 2 and Onsite 2 groups (1.1832, SE=0.5425) and a significant (t=2.181, p=0.0353) and small negative difference between the Onsite 2 group and the total effect (−0.3354, SE=0.1340) as well as a significant difference (t=−2.502, p=0.0149) with a low effect size (R2adjusted=0.125). In the Hollow_structure, the model detected a small negative difference between the Online 1 group and the total effect (−0.9830, SE=0.4386) and a significant difference (t=−2.241, p=0.0267) with a low effect size (R2adjusted=0.08). In the Solid_structure scores the model detected a small negative difference between the Onsite 2 group and the total effect (−1.23148, SE=0.43687) and a significant difference (t=−2.819, p=0.00558) with a low effect size (R2adjusted=0.025). In the Solid_structure_surface_distance the model detected a positive difference between the Online 2 and Onsite 1 groups (1.5026, SE = 0.5822) and a significant difference (t = 2.5809, p = 0.0113) with a low effect size (R2adjusted = 0.016). | yes |
| The effectiveness of a mobile application for the development of palpation and ultrasound imaging skills to supplement the traditional learning of physiotherapy students | Fernández-Lao et al. (2016) | single-blinded, randomized controlled study | physiotherapy students | 49 | NI | reported previous knowledge/training in musculoskeletal ultrasound imaging in a pre-enrollment questionnaire | hybrid | mobile application, as a supplement to traditional learning | all participants received theory and practice traditional training in ultrasound imaging and palpation of the musculoskeletal region of the shoulder in the same module of the study. The educational program had 6 contact learning lessons and 20 self-study hours that were focused on the theoretical and practical learning about palpation and ultrasound imaging procedures in the shoulder region. The students were randomly divided in two groups for the 2h theoretical lessons using a computer-generated number sequence. Teachers were blinded to the participant allocation group. Then these two groups were divided into two parts (2 groups in the experimental and 2 in the control group) for the 4h practical lessons to facilitate the learning process. The palpation skills sessions were conducted by the professors according to the method previously described and the ultrasound imaging sessions were developed according to a previously reported methodology. The 20 self-study hours were carried out by the students on their own using the mobile app or traditional study models (e.g., e-books, books, or journal papers available in the University Library), depending on the study group. The ultrasound device used by the participants was the same model for all participants. Both groups had two weeks to study after the oncampus session, but the m-learning group received this session after the control group completed the evaluation to avoid encouraging this group to seek information using the internet or accessing to the mobile app. The mobile application has specific content based on the specific region of the shoulder being studied. For each structure of the shoulder, there exists a theoretical description, a drawing with the anatomical description, an image with the specific placement of the ultrasound probe, an ultrasound slice, a diagram of the ultrasound image and a video of the manual palpation procedure. | experimental, with free access to the mobile application vs. control, with access to traditional learning materials on the topic | theoretical knowledge: MCQ with 20 questions; skills: OSCE with two components: ultrasound and palpation; quality of the intervention: 5-Point-Likert Scale; global satisfaction: rating on a NRS | ultrasound skills: statistically significant differences between the groups in the positioning of patient (p<0.001), where the experimental group showed better results compared with the control group. The group with the mobile app also positioned the ultrasound probe better than the control group did (p=0.007), and they demonstrated better handling of ultrasound probe (p=0.013). The differences between the groups in the orientation of the ultrasound probe (p=0.548) and the image adjustment (p=0.191) were not statistically significant. Palpation skills: the experimental group showed better positioning of the patient (p=0.009) and a better direction of palpation (p=0.021). They did not have significant differences in the position of the extremity (p=0.521) or the precision of palpation (p=0.116). OSCE: global scores were significantly higher in the experimental group than in the control group for both ultrasound (p<0.001) and palpation skills (p=0.034). There were no significant differences in the time required to perform the examination between the groups. Quality of intervention: higher ratings in the responses from the m-learning group compared with the control group for the items the teacher was competent (p<0.001), lessons were interesting (p=0.016), I was able to learn a lot (p=0.022), the size of the groups was optimal (p=0.002) and the teacher-student interaction was adequate (p=0.008). There were no significant differences between groups for the items theory and practice were well combined (p=0.114) and I would like to have been in another learning group (p=0.185). Global satisfaction: high ratings (8.200±0.767), on an 11 numeric point rating scale. | yes |
| ESCAPE-CARDIO: GAMIFICATION IN CARDIOVASCULAR PHYSIOTHERAPY. AN OBSERVATIONAL STUDY | Ferrer Sargues et al. (2021) | observational study | students of 3rd and 4th year of Degree in Physiotherapy | 58 (3rd + 4th course = experimental) / 37 (3rd course experimental) + 117 (3rd course control) | NI | 3rd course students were in the subject first exam call | hybrid | Educational Escape Rooms (EERs) | Three communicating classrooms were necessary. They were equipped with cameras and microphones, so the activity instructor could control the process from the monitoring room. A fourth room was proposed for the pre-game meeting. The material employed included locks of different types, a cryptex, test tubes, books, markers and ultraviolet flashlights and blood bags, among others. Finally, an introductory video was created by the CEU Audio-visual Media service. These videos are common in the ERs, and their purpose is to introduce participants to the story of the activity, achieving greater gamification and immersion in it. The students who accepted to participate were divided into groups of two or three people. Total time of sessions was 90 minutes. Students had to find clues when solving puzzles related to theoretical and practical concepts in the area of cardiorespiratory physiotherapy. Then they could move to another room until the last space, where they finally had to make a physiotherapy diagnosis based on the symptomatology of an advanced simulation mannequin. All the games were based on standardized physiotherapy and nursing interventions, and the design of the puzzles was based on a list of most characteristic puzzle types in recreational ERs worldwide. All the aspects that had been interesting to the students were discussed in a debriefing phase, focusing on the learning they took from the activity. | intervention group: exam of the cardiac rehabilitation subject at the end of the term; control group: their classmates, not participating in the activity, received a manual with the results of the clinical cases shown in the activity. | knowledge: marks obtained during the exam (whole 3rd course); qualitative evaluation of the activity (learning and applicability, integration in the teamwork model, assessment of the instructor’s work, gamification and activity immersion and overall assessment of the Escape-Cardio:3rd + 4th course): survey (5-Point-Likert-Scale) | knowledge: better performance in the 3rd EERs group, compared to the 117 of the 3rd NEERs group. Only one of the students who participated in the Escape-Cardio failed the final exam (3%), compared to a total of 40 students who failed to pass the course (30%). Likewise, of 11 students that not submitted to the exam (7% of the total), only one corresponded to a student who had completed the activity (3%). Average assessment mark of the exam in the 3rd EERs Group was 7.03±1.3 out of a maximum of 10, compared to an average qualification of 5.61±1.6 points of the students in the 3rd NEERs Group. Statistically significant difference between groups in the cardio-related section of the exam (p=0.00), since it was not for the other exam section (p=3.00); Survey: within the free text fields, most of the comments emphasized the applicability of the concepts that had been seen, the great immersion of the activity and the excellent group work, both with peers from their academic year and with students from another year. As negative aspects, it was only highlighted that it should not be a voluntary activity but integrated into the annual teaching plan. | yes |
| Doctor of Physical Therapy Education in a Hybrid Learning Environment: A Case Report | Gagnon et al. (2022) | case report | graduate cohorts of physiotherapy PhD Prorgam | 187 | NI | NI | hybrid | synchronous and asynchronous online learning + travel to campus twice per trimester for immersive blocks of in-person laboratory instruction | NI | NA | knowledge: enrollment and graduate outcomes, performance: Clinical Performance Instrument; clinical instructions: survey; student satisfaction: survey | Response rates for student and graduate surveys were 79% (n = 143) for the end of year 1 survey, 98% (n = 177) for the end of program survey, and 49% (n = 88) for the surveys administered 1 year after graduation. On student satisfaction surveys, 97% and 98% of students reported they were “very satisfied” or “somewhat satisfied” with their overall academic experience, respectively, at the end of year 1 and 2 in the DPT program. One year after graduation, 98% of graduates were “very satisfied” or “somewhat satisfied.” This is comparable with published national benchmarks for educational satisfaction in DPT students, which report 93.5% of United States DPT students “agree” or “strongly agree” that they satisfied with the quality of their DPT education.15 When asked to give the Baylor DPT program a “grade,” ranging from 0% to 100%, graduates assigned a mean “grade” of 90.34%. When surveyed during year 2 of the program, clinical instructors rated 84% of students as “prepared” or “well prepared” for their clinical experience and rated 19.2% of students as performing “significantly better” than students from other programs. One year after graduation, 20.81% of graduates reported they were enrolled in or had completed a residency, and another 14.6% of survey respondents reported they plan to complete a residency in the next 2 years. | yes |
| Physiotherapy students’ perspectives of online e-learning for interdisciplinary management of chronic health conditions: a qualitative study | Gardner et al. (2016) | qualitative study | Physiotherapy students | 23 | NI | NI | digital | Rheumatoid Arthritis for Physiotherapists e-Learning (RAP-eL) | Online e-learning platform designed for physiotherapists to increase their disease-related knowledge and clinical skills regarding the safe and effective management of rheumatoid arthritis (RA) within a chronic disease management framework. The purpose of the resource is to deliver contemporary evidence for physiotherapy management of RA (knowledge), and to integrate that information with practice-relevant clinical skills (skills). | NA | 3 focus groups: students’ perspectives on the advantages and disadvantages of online e-learning, ways to enhance e-learning, and information/ learning gaps in relation to interdisciplinary management of chronic health conditions, a semi-structured interview schedule was developed. | Three key themes were identified: 1. Preference for a combination of online e-learning and lecture-style learning modes given the advantages and disadvantages of each. 2. Using ‘real world’ clinical examples to bridge the gap between theory and practice. 3. Strategies to facilitate consolidation of e-learning. | yes and no |
| Meeting the Challenge of Instructor Shortages: A Blended Teaching and Learning Model for a Neuroscience Course in a Doctor of Physical Therapy Program. | Ge (2018) | retrospective nonexperimental observational study | doctor of physical therapy programme, second professional year | 36 | NI | NI | hybrid | blended teaching/learning | course: neuroscience; this basic science course links to patient/client management and differential diagnoses courses that address physical therapy evaluation and interventions for individuals who present with neurological movement disorders. Textbooks + supplementary material (journal articles, DVD, multiple online videos, customized flash video: all implemented in the LMS). Monthly face-to-face lectures + weekly onsite reviews + tutoring sessions. Assignements in this blended leaning model: reading, videos, games, quizzes, discussion board postings, 4 exams, active learning activities with 2 group presentations. | students who had traditional learning | perceptions: survey (18 Likert-Scale-Questions); knowledge: final grades | survey: overall outcome was positive. 91.6% strongly agreed/agreed that the content of the course was appropriate for learning neuroscience. 97.2% of the students strongly agreed/agreed that the LMS was well organized. 83.3% of the students strongly agreed/agreed that the course prepared them to be an adult learner capable of using distance learning eductaion delivery methods for future life-long-learning. Only 38.9% of students strongly agreed/agreed that the course was effective. 72.2% of the students participated in the open lab more than 5 times - but only 27.8% strongly agreed/agreed that the Jeopardy game facilitated their learning (36.1% for case presentation). Final grades: students who participated in the blended course performed slightly better (90.1% mean grade vs. 88.0%, with no significant difference: p=0.06). | yes |
| A STUDY ON SOME ASPECTS OF DISTANCE LEARNING FOR PHYSIOTHERAPY STUDENTS DURING A PANDEMIC | Gencheva and Gencheva-Vassileva (2020) | cross-sectional | physiotherapy students | 28: divided in two groups – 10 students in Physiotherapy in Pediatrics (practical course) and 18 students in the optional course of Fit-Ball. | NI | NI | digital | e-learning via Facebook groups and Viber groups | The main topics, the study materials, the student’s tasks given in the Facebook groups are discussed during the video call online classes. Different questions and teacher’s and students’ presentations were discussed through the video calls. In distance form of learning the emphasis is on students’ self-training. In regard to this every student had his own task. In the Fit-ball course the student’s task was to design a complex of exercises through complex of exercises trough pictures or video material of different starting positions, training of different muscle groups, case assignments for patients with various diseases and disabilities and combining exercises with and on large therapeutic balls using Thera Band tapes. During video calls the presented works were analyzed and individual errors were corrected. In the course of PT in pediatrics the task for independent work was to present written answers to special clinical questions on previously presented material (text or video file) on specific themes included in the course curriculum. Designing PT complex on previously presented clinical cases, preparation on abstracts, solving tests, etc. After the deadline for presenting the independent tasks they were presented and discussed during the group video call. | PT in pediatrics vs. PT Fit-Ball group | SWOT analysis: online questionnaire (specifics of distance learning, the chosen methods of communication, self-preparation and the difficulties encountered, specifics of the respective discipline) | SWOT: strength – experience in working with used platforms, no need in additional technical training, overcoming geographical boundaries, training can be conducted from anywhere in the world where there is access to the Internet, flexibility in determining the time for a conference call, in accordance with students and the teacher’s possibilities. Weaknesses – the students’ and teachers’ workload is bigger, lack of “face to face” contact which slows the communication process, plagiarism in the developing individual tasks, problems with internet connection prevent active participation in the learning process, investing funds for buying large exercise balls and elastic bands. Possibilities – participating in the learning process in case of illness, business trip, quarantine, more possibilities for selfpreparation and enlarging the knowledge in the field of study, the interactive nature of new technologies makes the learning process more interesting and easier for understanding. Threats – impossibility to actively include all students as it allows the presence in the study hall, no practice with patients, protection of the "intellectual property" of the developed teaching materials, strong dependence on modern technologies, updated operating systems, applications, compatible file formats, fully charged device battery, etc. | yes and no |
| Participation in Asynchronous Online Discussion Forums Does Improve Student Learning of Gross Anatomy | Green et al. (2014) | controlled trial | second-year anatomy course for physiotherapy students | 137 | NI | NI | hybrid | asynchronous online forums | Four to five hours of face-to-face teaching per week with approximately half being lectures and half being practical classes. Practical classes are cadaver-based using prosected specimens with some being clinical anatomy classes where students apply their anatomy to undertaking clinical assessment on fellow students. The course has a substantial LMS site that incorporates asynchronous online forums as a learning tool. Students were informed in their first class that online discussion forums are for student interaction and encouraged to post suggested answers. A typical thread is initiated by a student and responded to by other students. The weekly moderation by the course coordinator ensures that all threads are checked for accuracy of responses and to resolve unanswered questions. | comparison of two campus (Bendigo + Bundoora) | forum analysis: recording every students + staff who posted on that thread and total number of hits; indicator of academic ability: final mark + prerequisite mark | academic ability: campus had no effect on the final mark of students whether directly (MLE=-1.035, p=0.527) or indirectly through the total number of posts (MLE=0.830, p=0.154). Furthermore, there was no total effect of campus on a student’s final mark (MLE=-0.206, p=0.864). Forum analysis: the direct effect of campus on the total number of posts appears large (MLE=4.424), suggesting that Bendigo students on average place 4.424 postings more than Bundoora students. Due to large standard error of the MLE of this direct effect, there is not enough evidence to suggest that there is a direct effect of campus on total number of posts (p=0.073). The prerequisite mark of a student had a direct positive effect on the total number of posts (MLE=0.336, p=0.001). This suggests that for every 1 mark increase in prerequisite mark, the total number of posts by a student increased on average by 0.336 posts. The total number of posts also had a positive direct effect on the final mark for a student (MLE=0.188, p=0.008). For every additional student post, a student’s final mark increased on average by 0.188 marks. Finally, the prerequisite mark for a student impacts on their final mark both directly (MLE=0.646, p=0.002) and indirectly through the total number of posts (MLE=0.063, p=0.003), resulting in total effect of prerequisite marks on a course final mark (MLE=0.709, p=0.002). This suggests that (1) for every 1 mark increase in prerequisite mark, the final mark for a student on average increased by 0.646 marks and (2) for every 1 mark increase in prerequisite mark, the final mark for a student increased on average by 0.063 because of the effect that a student’s prerequisite mark had on a student’s total number of posts. Discussion forums were the most used feature of the LMS site with 31,920 hits and 48% of all students posted at least once with a higher proportion of Bendigo (56%) than Bundoora (44%) students posting. Over the course of the semester, there were 190 threads of which only four were initiated by staff and a total of 608 posts by students and 313 posts by staff. The number of posts per thread ranged from 1 to 19 with a mean of 4.8. | yes |
| Impact of Introduction of Blended Learning in Gross Anatomy on Student Outcomes | Green and Whitburn (2016) | retrospective cohort study | physiotherapy students | 461 | NI | NI | hybrid | blended learning; 2013: video conference to combine the campi; 2014: face-to-face lectures were partly replaced with online content; 2015: face-to-face lectures again reduced. digital content: short online video clips, asynchronous online discussion forums. | course: anatomy, covering regional anatomy of the vertebral column, lower limb and thorax. 2013: course involved approximately five hours of face-to-face teaching per week for 13 weeks with approximately two hours of lectures (25 for the semester), a two hour practical class centered on prosected cadaveric specimens but including other anatomical materials such as x-rays, plastinated cadaveric sections and models and, in most weeks, a one hour clinical anatomy practical that introduced students to clinical skills such as palpation and clinical testing. Instructor to student ratios in practical classes vary slightly, but are normally in the range of 1:25. Lectures deliberately include ‘buzz groups’ where questions are presented and after a short discussion in their groups, students report back to the whole lecture. Particular attention is paid to directing questions, and expecting answers, from both campuses via the video conference with a staff member at each campus to relay responses via the microphone. 2014: semester length was reduced to 12 weeks. Some face-to-face lectures were replaced with online content (reduced to 16 lectures total). Practical-based aspects were retained in the blending process. Practical classes remained largely the same but reduced proportionally for the 12 week semester. 3 tutorial classes were introduced, timed to occur before practical tests and used a question and answer format where students could raise issues that they were having difficulty understanding. 2015: identical course learning outcomes and practical classes. Content was delivered with a deliberately blended learning strategy of one lecture per week (total of ten for the semester) and remaining content delivered using a series of short online video clips. The videos were supported by asynchronous online discussion forums that had already been integrated into the course. While watching online content a student may be asked to pause the video and complete a ‘fill in the blanks’ task on an online worksheet to check comprehension. In this case, the answers may be presented subsequently in the online content whereas for other activities the student may be directed to discuss the answers to a worksheet activity on online discussion forums with their peers. | three different cohorts (2013, 2014, 2015) | academic ability: pre-requisite marks (to enscure cohort-similarity at baseline); student knowledge: aggregate practical test mark + examination mark; level of engagement with the course: number of student posts on discussion forums (students were classified as non-contributors (no posts on discussion forums), low level contributors (0-9 posts for semester) and high level contributors (>10 posts for semester); student perceptions of the course: standardized questions on the university Student Feedback on Subjects (SFS) process (Likert-Scalen, open ended) | academic ability: there were no differences in the mean marks for the prerequisite anatomy course between the three years (F(2,375)=1.35, p=0.26). Knowledge: differences in mean mark for practical tests (F(2,459)=37.26, p<0.00) and the final examination (F(2,455)=10.86, p<0.00) between cohorts. Post-hoc tests revealed that the mean marks for 2013 were lower than both 2014 and 2015 for both practical tests and the written examination. Perception: for most items there were no differences between cohorts. However, there were significant differences for level of intellectual stimulation (H(2)=10.897, p=0.004) and appropriateness of the amount of work required (H(2)=9.564, p=0.008). Post-hoc comparisons revealed that the 2014 cohort reported lower levels of intellectual stimulation than both 2013 and 2015 cohorts. The 2015 cohort were less likely to report that the amount of work that was required was appropriate than both the 2013 and 2014 cohorts. Level of engagement: the proportion of non-posting, low and high-posting students on LMS discussion forums did not change across the 3 years of the study (x2(4)=5.13, p=0.27). | knowledge: yes; perception: no |
| The Relationship between Student Engagement with Online Content and Achievement in a Blended Learning Anatomy Course | Green et al. (2017) | retrospective cohort study | students from from Physiotherapy (PT), Exercise Physiology (EP), and Exercise Science (ES) programs | 500 (311 physiotherapy students) | NI | NI | hybrid | blended learning | 12-week course consisted of: one face-to-face interactive lecture per week (total of 10 for the semester), 74 short online video clips, 24 hours of practical class (principally prosected cadaveric specimens but including other anatomical materials such as X-rays, plastinated cadaveric sections and models), 8 hours of clinical anatomy practicals that introduced students to clinical skills such as palpation and clinical testing, and 3 hours of tutorials. The online videos are 5–10 minutes in length and replaced approximately 14 hours of previous face-to-face lectures. Students are asked to complete online content by a set date (timed to ensure content is covered before practical classes) while using an accompanying worksheet prepared for each series of videos. Online videos are typically interactive so a student may be asked to pause the video and complete a “fill in the blanks” task on the worksheet to check comprehension. In this case, the answers may be presented subsequently in the online content whereas for other activities the student may be directed to discuss the answers to a worksheet activity on online discussion forums with their peers. The LMS site also contained a range of resources including links to the 74 video segments (75 in 2016) and six asynchronous online discussion forums (one for each of the 3 regional anatomy modules, one for assessment, one for general enquiries and one for staff announcements). | Students were divided into four groups for analysis based on campus and program of enrolment; (1) Bendigo PT, (2) Bendigo EP/ ES, (3) Bundoora PT, and (4) Bundoora EP/ES. | students use of online material: proportion of the text-based documents viewed, proportion of video segments viewed and the total number of hits on the online discussion forums; incoming academic ability: prerequisite mark; students knowledge: final mark (50% of the final grade with a final written examination making up the remaining 50%) + in-semester assessments (3 flag-race style practical tests) | knowledge: students in the PT program scored higher on final course grade (F(1,496)=301.2, p<0.001) and prerequisite course grade (F(1,433)=127.0, p<0.001) than ES and EP students. Students use of material: PT were also more likely to view a higher proportion of LMS videos (F(1,496)=106.8, p<0.001), LMS documents (F(1,496)=76.9, p<0.001), and had more hits on online discussion forums (F(1,496)=29.6, p<0.001). Second level analysis: the squared multiple correlation (R2) for course final grade indicate that its predictors (prerequisite final grade plus the two mediators) explain 32% of its total variability. The direct (unmediated) effect of prerequisite grade on course final grade implies that a 1% increase in prerequisite grade yields an average increase of 0.639% in course final grade. The total effect (direct plus mediated) on course grade is 0.639 + 0.086 + 0.028 = 0.753 so that a 1% increase in prerequisite grade mediated by percent videos and online discussion forums viewed implies an average increase in final grade of 0.764%. The ratio of mediated effects to the total effect of prerequisite grade ((0.086 + 0.028)/0.753) suggests online activities appear to enhance its effect by around 15%. The model also indicates that students with stronger academic performance on the prerequisite course were more likely to watch the videos on the LMS site (regression weight = 0.441, p<0.001) and had more hits on the discussion forums (4.033, p<0.0001). | yes |
| A massive open online course (MOOC) can be used to teach physiotherapy students about spinal cord injuries: a randomised trial | Hossain et al. (2014) | randomised parallel controlled trial | Second-year and third-year undergraduate physiotherapy students | 48 | (1) over 16 years of age, (2) willing to participate, (3) regular Internet access | insufficient English to provide consent, and complete the online modules and assessments | digital | Massive open online courses (MOOCs) | Experimental group: 5-week MOOC titled Physiotherapy Management of Spinal Cord Injuries, 3 hours per week. Course curriculum, objectives and a weekly study plan were provided. Pre-course and post-course quiz invitation. At the beginning of each week, the EG participants were emailed three to six tasks to complete. By the end of the course, the EG moved through the 14 physiotherapy-specific lessons. They were also required to do some additional reading and engage in an online Facebook discussion for all MOOC registrants. Checks were made to ensure that all of them joined the Facebook group. The MOOC did involve viewing short videos from two teachers at the beginning of the course and then from one teacher each week. The videos outlined the content of the course and learning material for each week. Learning content included Interactive screens that require students to regularly stop, think and perform a learning task. The interactive screens require students to enter text in response to questions, view videos and analyse movement through multiple-choice questions, select appropriate exercises for particular problems, and constantly reflect on content learnt through drop-and-drag activities, matching exercises and various other interactive tasks. In addition, there are over 150 videos of people with spinal cord injuries, and interviews with both physiotherapists and patients from a diverse range of countries. | The control participants were asked to move at their own pace through the physiotherapy-specific module of the MOOC over a 5-week period. They were instructed to devote 3 hours per week to their studies as well. Checks were made to ensure that none of the control participants registered for the MOOC or joined the MOOC discussion forum run through Facebook. | knowledge: two 20-item multiple-choice tests were devised specifically for this trial; perceived confidence to treat people with spinal cord injuries and satisfaction with the learning experience: series of questions or statements that required students to respond on a scale from 0 to 10 | knowledge: the mean between-group difference was 0.7 points (95% CI –1.3 to 2.6) on a scale from 0 to 20, with a positive score favouring the EG. Perceived confidence and satisfaction with the learning experience: mean group difference was 0.4 points (95% CI –1.0 to 1.8) and 0.0 points (95% CI –1.1 to 1.2), respectively on a scale from 0 to 10, with a positive score favouring the EG | not clear |
| Simulation-based education improves student self-efficacy in physiotherapy assessment and management of paediatric patients | Hough et al. (2019) | prospective, observational study | physiotherapy students | 92 from cohort of 2018; 74 from cohort of 2014 | enrolled in the Paediatric Physiotherapy Practice academic unit | Students who had previously attended any classes in the Paediatric Physiotherapy Practice unit (or equivalent) | hybrid | simulation-based education (SBE) with high fidelity mannequins + eLearning | The scenario for each SBE session specifically targeted one of the primary clinical domains of paediatric physiotherapy and was delivered with the corresponding musculoskeletal, cardiorespiratory and neurodevelopmental modules of the unit. In the week prior to the SBE session, the students were requested to independently undertake an eLearning package to prepare them for each session. The interactive SBE sessions used high fidelity paediatric human patient simulators which are life-like, anatomically correct, computer driven mannequins with physiologic responses that mimic real patients. Each SBE learning scenario was conducted over a two-hour period with 25 students per group. | two different cohorts: 2014 (the first year of simulation delivery) and in the exact same format in 2018 (the most recent year to determine if there was evidence for ongoing delivery of SBE) | student self-efficacy in the physiotherapy assessment and management of paediatric patients: self-efficacy questionnaire (5-point Likert scale; at the first 5min of each session and at completion of the SBE scenario); student satisfaction with SBE: learning reactionnaire (5-point Likert scale, option to add comments) | self-efficacy: Significant increases in mean student self-efficacy were recorded for all questions in each scenario (p < 0.001) with improvements in mean self-efficacy scores ranging from 0.73–0.97 for all questions across all scenarios / stion 2 (clinical decision-making) had the largest mean improvement to self-efficacy in 2 out of the 3 scenarios (musculoskeletal and cardiorespiratory) as well as the largest mean improvement overall (0.93), while question 4 (maintaining communication with nurse, carer and child) had the lowest mean improvement in 2 out of the 3 scenarios (musculoskeletal and cardiorespiratory) as well as the lowest mean improvement overall (0.77). Questions 4 and 7 had the highest pre and post SBE scores in all 3 scenarios, and respectively had the highest pre (1.99, 1.91) and post (2.76, 2.72) scores overall. Question 2 had the lowest pre and post SBE scores in every scenario and overall (pre = 1.48, post = 2.41). / satisfaction: Most student responses (78.6%) indicated that the SBE scenarios were an effective model for promoting learning in the field of paediatric physiotherapy. - comments: primary theme identified was experience was mentioned alongside the concepts of skills, practical, helped, information, and simulated | yes |
| Using video podcasting to enhance the learning of clinical skills: A qualitative study of physiotherapy students' experiences | Hurst (2016) | qualitative exploratory study | physiotherapy students | 31 | NI | NI | hybrid | video podcasts | technology enhanced learning of clinical skills with the help of video podcasts | four different focus groups: (1) first year pre-registration physiotherapy students (n =9); (2) second year pre-registration physiotherapy students (n =6); (3) second year undergraduate physiotherapy students (n =8); (4) third year undergraduate physiotherapy students (n =8) | experiences: four, one-hour long focus groups | seven themes for usage of vodcasts to help clinical skill development: revision, developing a step by step process, repetition, refinement of skills, confirmation/comparison, authenticity/quality, placement benefits. Experiences of using vodcasts and the development of skill acquisition: students overwhelmingly agreed that the vodcasts allowed them to develop their technical ability to perform a skill. Students all agreed that some of the current vodcasts were better than others in helping the students learning of procedural knowledge. Using the vodcasts wasn't perceived as the main way to develop clinical reasoning. Students felt that the vodcasts affected their self-efficacy in a positive way. Sequence of key activities to enhance the acquisition of clinical skills: watching the vodcasts before, during and after classes were all identified as important in the acquisition of clinical skills. Preferred learning environment: type of learning environment that best engaged the students to learn skills supports a blended, multisensory approach. Students reported the value of tutor feedback, correction and positive reinforcement through taught skills classes. They overwhelmingly agreed that it was best to learn a skill though a combination of different methods. | yes |
| Use of vision-based augmented reality to improve student learning of the spine and spinal deformities. An exploratory study | Kandasamy et al. (2021) | exploratory study; post-intervention crossover design | final year bachelor degree physiotherapy students | 74 | NI | NI | hybrid | Vision-based augmented reality (VBAR) mobile application | Task (1) involved learning the anatomy of the spine. Task (2) involved learning the pathologies of the spine using both traditional and VBAR learning (two groups) | traditional learning | evaluation of VBAR as educational tool (level of understanding): questionnaire (8-Likert-questions; 1 open ended question) | evaluation: significant difference between the two groups in all questions (p≤0,05) pro VBAR | yes |
| Being knowledge, power and profession subordinates: students' perceptions of Twitter for learning | Lackovic et al. (2016) | qualitative study | undergraduate (bachelor) physiotherapy degree students | 43 participated, 2x12 students in the focus groups | NI | NI | hybrid | twitter entries | A module-specific hashtag (#EBP2) was created. In addition to the regular lectures and seminars, discussions under the hashtag were encouraged (questions/comments/sharing of information and links) for a period of eight weeks. Following each weekly lecture, an #EBP2-tagged question relating to that lecture was tweeted for students to respond to. This question was tweeted by the contributing teacher-researcher. A few times at the end of the lecture, the tutor/teacher encouraged students to tweet. He observed any subsequent Twitter activity and would contribute to conversations linked to #EBP2. The students were advised to ‘follow’ the tutor who tweeted questions, and to ‘follow’ each other for 8 weeks. | NA | baseline questionnaire: use of social media and portable devices, distinguishing between use for personal reasons (e.g. entertainment) and for academic learning purposes (e.g., learning something related to their module, course or general interest within the profession); thoughts about Twitter usage (enablers, obstacles): two focus groups | At the end of the eight weeks of hashtag intervention, there was only one student tweet. Focus groups: themes explored were (1) Twitter obstacles to student contribution as “technology use obstacles” and (2) “obstacles related to the perception of Twitter”, the latter theme gathered around the concepts of “career/profession” and “celebrification”. A list of “enablers” was also identified, that is, what most students thought and/or agreed would encourage them to use Twitter for learning. | no |
| Teaching Physiotherapy Students Physical Examination Skills by Using Photogrammetry | Lo et al. (2022) | randomized controlled trial | first-year physiotherapy bachelor honor degree students | 77 | (1) first-year students, (2) no experience in manual skills on the cervical spine (3) willing to participate in the study. | physical/ mental conditions that would hinder their performance | digital | online course with 3D images for manual assessment skills | training on manual assessment skills in a 2h online course for learning 3 cervical spine examination skills in 2 homogeneous standard classrooms . 4 classes on an online learning platform were conducted with approximately 19 to 21 students per class. These 4 classes were monitored by 2 non-teaching staff, who distributed materials and ensured that the students could not access additional materials. Experimental group: 3D images of the manual therapy techniques | Control group: similar materials, but all images were 2D, including 2 pictures for each skill from the front and side perspectives, which is consistent with what they would receive in a traditional setting | knowledge: OSCE mark from the last trimester as reference score + OSCE mark after online course | knowledge: the participants in the 3D image group had significantly higher overall OSCE scores (mean=41.3 and SD=3.5) as compared with participants who were learning by using the 2D images (mean=39.1 and SD=4.5). t(75) value of −2.4 (p=0.02) with a medium effect size of d=0.54, | yes |
| The Ecofisio Mobile App for Assessment and Diagnosis Using Ultrasound Imaging for Undergraduate Health Science Students: Multicenter Randomized Controlled Trial | Lozano-Lozano et al. (2020) | double-blinded, randomized controlled multicenter study | undergraduate physical therapy students | 105 | (1) not having received any previous training in ultrasound imaging and management, (2) having anatomy and biomechanics knowledge, (3) being enrolled in any required subjects, (4) having the basic ability to use a mobile app, (5) having a mobile phone with Internet access running on the Android operating system | NI | digital | Ecofisio mobile app: focused on sports pathologies and contains relevant written and digital information about ultrasound imaging and management, as well as diagnosis of sport injuries. For each anatomical structure, there exists a theoretical description, a drawing with the anatomical description, an image with the specific placement of the ultrasound probe, an ultrasound slice, a diagram of the ultrasound image, and a video of the manual palpation procedure. | six contact sessions on site; this was followed by self-study time focused on the theoretical and practical learning of ultrasound imaging procedures, on different areas of the body, regarding sports pathologies. Each study group (Ecofisio intervention group and CG) attended 2 hours of theoretical lessons and 4 hours of practical lessons (ultrasound imaging). Finally, there was a 2-week self-study period, where the Ecofisio group used the mobile app and the CG used traditional study models (e.g., books and journal papers) | tranditional learning in the second self-study period | practical knowledge: OSCE (participants’ hands-on skills in terms of ultrasound management); theoretical knowledge: MCQ (20 questions); satisfaction on interventions: survey; satisfaction on app usage: survey (only app group) | theoretical knowledge: significant difference in the theoretical exam score (p<0.001) in favor of the Ecofisio group: mean 7.3 (SD 1.5) points. Practical knowledge: significant differences in all components assessed during the OSCE stations (p<0.001 for all) in favor for the Ecofisio group. Significant differences between groups in terms of passing the exam in favor of the Ecofisio group (43/50, 86%, vs 15/55, 27%, p<0.001). Satisfaction: Ecofisio group showed a higher satisfaction level than the control group for the following items: the teacher was competent (mean score 4.8 [SD 0.4] vs 4.5 [SD 0.5], p=0.009) and I believe that the training is applicable (mean score 3.3 [SD 0.8] vs 3.0 [SD 0.9] | yes |
| Effectiveness of online teaching during the COVID-19 pandemic on practical manual therapy skills of undergraduate physiotherapy students | Luedtke et al. (2022) | part A: cross-sectional; part B: longitudinal randomized controlled design | physiotherapy students: 1st year had classroom teaching, 2nd year had online teaching, 3rd year had classroom teaching | part A: 63; part B: 56 | enrolled at the Bachelor of Science program in physiotherapy at the University of Luebeck | NI | digital | video-based teaching | Part A: two manual therapy tasks to perform (instructions written and attached to the wall), that were chosen since they are standard techniques which were taught during classes (online and classroom) and include the treatment of both peripheral and spinal joints. The task had to be performed on a mock patient and were filmed. Part B: after practicing manual therapy techniques during the summer school for 4–5 hours, the students drew a raffle ticket which randomized them to learn a new technique (upper cervical quadrant [18]) either from a lecturer and practice the technique on a peer student or to learn the same technique from a video which was recorded by the same lecturer and to practice the technique on a lifesized rescue dummy. The lecturer was unknown to the students. After practicing the new technique for 3–4 times, students were video-recorded individually while performing this new technique on the same mock patient as used for part A. | traditional teaching | part A: perceived effectiveness of the manual therapy classes: 11-point Likert scale; general learning experience wih online material: 11-point Likert scale; part A + B: students’ performance: rated by two independent researchers based on the video material | performance: - part A: the most advanced group (third year), who had learned manual therapy in the classroom, received the highest scores but there was no significant main effect for the cohorts. Results for the lumbar spine technique were F(2,59)=2.271; p=0.112; ηp2=0.071 and for the technique on the knee joint F(2,59)=3.028; p=0.056; ηp2=0.093. - part B: better performance of the lecturer group after correction for duration of study F(1,51)=15.045; p<0.001; ηp2=0.228. | no |
| Transforming traditional physiotherapy hands-on skills teaching into video-based learning | Luginbuehl et al. (2023) | cross-sectional observation | undergraduate physiotherapy students | 28 (answering questionnaire A) + 24 (answering questionnaire B) | NI | NI | hybrid | technology-enhanced learning concept: on-site video-based psychomotor skills learning concept, which is embedded in asynchronous e-learning sessions that teach the theoretical background of applied psychomotor skills | course: complex precongestive physiotherapy lessons (6 e-learning lessons and 12 on-site video-based hands-on skills lessons); content: evaluation and management of edema following trauma or orthopedic surgery, and chronic lymphedema, e.g., upper extremity lymph edema, following breast cancer surgery and/or radiation; short theory inputs (screencasts) which included student activities (e.g., practice questions, processing of patient case examples, quizzes, etc.), formative test questions, and a discussion forum. They were complemented by practical assignments such as self-bandaging or edema assessments. E-learning was provided via LMS. Hands-on skills learning-material for these teaching topics was upgraded using some already available short silent videos, to which verbal explanations and written comments were added. Additionally, new videos were produced. To facilitate on-site learning, students watched the provided instructional videos in pairs and followed a checklist guiding them through the respective tasks, which they then practiced on each other. A circulating faculty member provided individual feedback and answered any arising questions. | NA | evaluation of the learning concept: questionnaire A (5-point-Likert-Scale); preferences, advantages, future improvements: questionnaire B (single-choice + open questions) | evaluation of the learning concept: students rated the teaching quality of this new concept between 1.5 ± 0.5 and 1.8± 0.4 on the Likert scale from − 2 to + 2. Preferences, advantages, future improvements: seven students (29.2%) preferred the traditional teaching concept while 16 (66.7%) preferred the new videobased learning concept of hands-on psychomotor skills. Open questions: students appreciated that the videos were available to them at any time, which allowed easy review and repetitive practice, e.g., prior to exams. They appreciated the ability to learn remotely and review the videos during practical learning sessions. Described benefits of the video-based skills learning concept were that it fostered self-paced and self-directed learning and provided all students with the same and equal good view of the skills demonstrations. However, students believe that a live demonstration at the beginning of the skills lesson would enable them to better judge details of a skill procedure, e.g., the amount of force needed for successfully completing a skill. | yes |
| Have we failed them? Online learning self‐efficacy of physiotherapy students during COVID‐19 pandemic | Madi et al. (2023) | mixed methods online survey | undergraduate physiotherapy students | quantitative data: 209 (P1) to 578 (P2) ; qualitative data: 158 (P1) to 457 (P2) | NI | NI | digital | online learning | not further explained | NA | all assessed at two timepoints (P1, P2) with 12 month in between; knowledge: grade point average; learning in online environment, time management, use of technology: online learning self‐efficacy scale (OLSES) ; students' self‐efficacy in relation to their perception of academic success: academic self‐efficacy scale (ASES); factors that augmented or reduced the perceived level of self‐efficacy: free entry boxes | knowledge: there was a significant difference between students interms of their GPA, X2(4)=46.301, p<0.01, with higher mean ranks at P2. Academic self-efficacy: significant difference in ASE scores between P1 and P2 with lower score at P2 (t=3.960, p<0.001, 95%CI:1.792–5.317). Online-learning self-efficacy: significant difference was found between P1 and P2 with a lower score at P2 in OLSE (t=6.043, p<0.001, 95%CI:8.086–15.867). Statistically significant decrease in the perceived level of digital abilities (U=51,594, p<0.01), the level of approval of online learning (U=41,003, p<0.01), and the likelihood of signing up for future online courses (U=40,664, p<0.01). Second level analysis: significant weak correlation between students' GPA and their OLSE (r=0.094, p<0.01) as well as their ASES (r=0.316, p<0.01) at P2. Qualitative data: shift in students' view of online learning, with the majority expressing dissatisfaction at P2 of data collection. (1) need to develop their online learning skills and demonstrated a heightened sense of responsibility, (2) motivation declined because of expressed mental distress, multiple distractors, limited social interaction, and an unfavourable teaching format, (3) availability of university‐paid Internet access and designated online learning platforms facilitated online learning, especially the ability to repeat recorded lectures, (4) ducators underutilised theseplatforms, (5) staying home saved travel time, (6) sense of injustice because of cheating. | yes (knowledge), no (rest) |
| Preparing undergraduate students for clinical work in a complex environment: evaluation of an e-learning module on physiotherapy in the intensive care unit | Major et al. (2020) | A mixed methods proof of concept study | undergraduate (3rd + 4th year) physiotherapy students | pilot phase: 7 students, 2 experts; main study: 7 students, 3 experts | NI | NI | digital | e-learning module with a variety of teaching materials: interactive assignments, background literature and short quizzes, self-developed and online videos (patient-testimonials, patient and PT observations, skills-modelling videos), and text presented in presentation slide format + online community as support | course: Physiotherapy in the ICU. The e-Learning module enabled students to anticipate clinical situations and PT tasks in the ICU. The course was implemented in the curriculum of the academic year 2016–2017 for 2x 6 weeks. | NA | Data collection Phase 1+2: focus group (semi structured) interviews for 30-60min; MCQ (25 questions) on obtained knowledge and reasoning skills, user-friendliness, level of complexity, didactic alignment and accessibility | Data collection Phase 1: the content of the e-learning module was perceived positively with regards to: accessibility, degree of difficulty, variation in study assignments, encouragement of further learning and triggering curiosity, and transparency of the module’s learning objectives. MCQ: only 11 out of 25 questions corresponded with the course objectives. Phase 2: The following themes were identified: expected competencies of PT students in ICU, feeling prepared for ICU clinical work and dealing with local variety. | yes |
| The effect of student self-video of performance on clinical skill competency: a randomised controlled trial | Maloney et al. (2013a) | single centre, randomised, double blind, controlled trial | 3rd year physiotherapy bachelor students | 60 | NI | NI | digital | video-based learning | self-produced video of the students doing clinical examinations were uploaded on the LMS. Group feeedback on weakness/strength of observed in the performance + formal reflective component where students were asked to compare and contrast their own performance against a peer benchmark performance were performed. In week eight of the semester, the students were randomly allocated into two groups. A key skill delivered to both groups in the regular practical classes of week 8 was assessment of an acute cervical spine condition. The intervention group **(**50%) were asked to supplement the teaching of this skill by completing a self-video of performance submission relating to this skill (Skill A). | Control group**:** completed their self-video of performance submission relating to an alternate skill less relevant to the cervical spine (upper limb neurological assessment). | performance of skill: OSCE grades; student perceptions and experiences relating to the teaching method: paper-based questionnaire (5-point Likert Scale, open ended questions) | performance: mean scores for the performance of Skill A were significantly higher for the intervention group (mean (sd) intervention group; 21.22 (4.24), control group; 18.96 (3.95), p=0.048); this difference in group means corresponds to a 7.5% increase in grades for the OSCE examination station. Alternate OSCE station skill results returned no significant difference in group results [mean (sd) ‘intervention group’; 17.26 (6.27), ‘control group; 19.78 (4.36), p=0.093]. Perceptions and experiences: all students indicated agreement (agree/strongly agree) to the statement ‘reflecting on your self-video performance allowed you to notice areas for improvement in your mannerisms and communication’; majority of students agreed/strongly agreed, with the statement that reviewing the submission from a peer (‘peer-benchmarking’) was helpful in developing their clinical skill level. | yes |
| Investigating the efficacy of practical skill teaching: a pilot-study comparing three educational methods | Maloney et al. (2013b) | randomized controlled trial | 3rd year undergraduate physiotherapy students | 40 | NI | NI | digital | (1) pre-recorded video tutoring (PVT): 30min; content: demonstration of the skill being applied to a patient actor before the tutor repeating the skill with commentary to allow illustration of the tutor’s clinical reasoning. Text prompts act as a trigger for student problem solving, and instructed the students to conduct 10 min of practise with a student colleague. (2) student produced self-video (SSV): students were given a written simulated patient scenario via the LMS. Scenario was constructed so that effective management of the patient in the scenario required the student to utilise the practical skill of interest. Key elements: (2.1) source the knowledge to complete the skill required within the patient scenario, (2.2) produce a 5 min film of themselves as therapists managing the patient in the scenario, (2.3) submit the video to the LMS where a remote tutor was able to review the submission and give brief written feedback, (2.4) view an exemplar peer video as selected by the tutor to optimise bench-marking of expected performance level, (2.5) complete a brief written reflection contrasting their own performance to the peer exemplar. | Each practical group was exposed to one of three practical skill teaching methods utilised to teach two complex clinical skills taught throughout the semester. **Skill A** (week 2): initial assessment and subsequent patient education of an acute, and highly irritable, cervical spine complaint; outpatient was depicted as anxious about their condition and prognosis, was resistant to being handled by the physiotherapist, and had been discontent with previous physiotherapy treatment; s**kill B** (week 8): initial assessment and treatment of a vestibular inpatient, experiencing distressing levels of dizziness with movement; inpatient setting, client in bed, trying to remain very still to prevent any aggravation of the symptoms. | traditional tutoring (TRAD): live demonstration of the entire skill provided by the practical class tutor; discussion followed of the skills individual elements and how these elements might require tailoring to the individual patient; 30min | questionnaire: participants level of satisfaction with the teaching method + perception of utility + teaching methods educational value; clinical skill performance: OSCE | performance: mean scores for the performance of skill A and B were comparable between group 1 (TRAD, n=15), group 2 (PVT, n=16) and group 3 (SSV, n=13), with no statistically significant difference detected for the effect of the three teaching methods on clinical performance in the OSCE. Perceived educational values: significantly higher Likert scale ratings within the PVT (p=0.003) and SSV (p=0.007) groups in comparison to TRAD, whilst comparisons between the PVT and SSV groups were equivalent (p=0.80); ratings of learner satisfaction were comparable between the three groups. | performance: no; preceived educational value: yes |
| Evaluation of a multimedia online tool for teaching bronchial hygiene to physical therapy students | Marques da Silva et al. (2012) | randomized controlled trial | 4th year undergraduate physical therapy students | 16 | NI | NI | digital | "cursos on-line": multimedia online tool for teaching | course: bronchial hygiene techniques (BHT); each module consisted of a theoretical component (booklet), multimedia resources (figures, videos, and graphic animations), and interactive components (discussion list among instructors and students, a system with online tests, and links to selected information of other websites). The booklets containing evidence-based information was based on Medline and EMBASE databases. Length of the videos: ca. 3min. Some videos were accompanied by animation sequences illustrating physiopathological conditions. External links were used to guide the students to further references. Both groups attended the conventional course classes. After the end of the course both groups had access to the teachers to ask questions and to study, respectively, their online or conventional material along two weeks. | conventional written material for the BHT modul | knowledge: initial supervised MCQ + final MCQ (20 questions, 10-Point NRS) | knowledge: on the final test, the students in the online group performed significantly better than the control group (7.75 (±1.28) and 5.93 (±0.72), respectively; p<0.05). | yes |
| Effectiveness of a heart disease blended learning program in physiotherapy students: a prospective study | Marques-Sule et al. (2023a) | prospective study | 2nd year undergraduate physiotherapy students | 108 | NI | previous heart disease training | hybrid | blended learning (BL): asynchronous online learning modules and scheduled online activities | topic: cardiac pathology; 8-week BL program; asynchronous online learning modules and scheduled online activities with face-to-face lectures offered at set points during the semester. Participants had autonomy and flexibility for accessing online course content, except for the seven scheduled face-to-face classes. The online resources used were as follows: (1) LMS was utilized as a virtual communication platform; (2) online syllabus about heart disease composed of seven themes developed from the knowledge considered as basic for health allied students; (3) online videos of international and national scientific societies + one-minute bullet-point videos; (4) links to websites of international and national scientific societies; (5) podcasts of international and national scientific societies; (6) online multiple-choice questionnaires; (7) mobile apps; (8) forums, emails and online tutoring were delivered | NA | Knowledge acquisition of cardiovascular risk factors and cardiovascular disease: “Coronary Artery Disease Education Questionnaire” (CADEQ) + “Effect of the Dader Method in Cardiovascular Risk of Patients with Risk Factors or Cardiovascular Disease Questionnaire” (EMDADER); motivation: Academic Motivation Scale and Attributional Styles Questionnaire; engagement: Student Engagement Questionnaire; design of the program instructions: Community of Inquiry Survey; learning behaviours: number of downloads, topics visited and total score of questionnaires collected from LMS | knowledge: - CADEQ score: significant interaction between intervention and measurement times (F(4,304)=11.63, p<0.001, η2=42.92). A gradual and significant increase of knowledge with respect to T1 was observed in pairwise comparisons in the T2 assessments (mean difference = −0.87 ± 0.26), p=0.02, Cohen’s d=−0.19), in T3 (mean difference = −1.296 ± 0.29), p<0.001, Cohen’s d=−0.54), in T4 (mean difference = −1.659 ± 0.25), p<0.001, Cohen’s d=−0.66) and T5 (mean difference = −1.13 ± 0.285), p=0.001, Cohen’s d=−0.48). Furthermore, a higher score was observed after one-month follow-up than mid-program (mean difference = −0.78 ± 0.23), p=0.01, Cohen’s d=−0.41). - EMDADER scores, there was a significant interaction between the intervention and the measurement times (F(4,324)=13.88, p<0.001, η2=55.51). In addition, the knowledge gained increased significantly with respect to T1 in all subsequent assessments: T2 (mean difference = −0.89 ± 0.19), p<0.001, Cohen’s d=−0.75), T3 (mean difference = −0.56 ± 0.16), p=0.01, Cohen’s d=−0.53), T4 (mean difference = −1.10 ± 0.20), p<0.001, Cohen’s d=−0.94) and T5 (mean difference = −1.05 ± 0.17), p<0.001, Cohen’s d=−0.90). Significant differences were further observed between T3 and T4 measurements (mean difference = −0.54 ± 0.17), p=0.02, Cohen’s d=−0.50) and T5 (mean difference = −0.49 ± 0.14), p=0.01, Cohen’s d=−0.51). Motivation: similar scores at the T2 and T3 assessments (p>0.05). Engagement: after the intervention, 87.04% agreed or completely agreed with the items of the Teaching dimension; 81.48% with the items of the Intellectual Capabilities dimension; 78.70% with the Teacher-Student Relationship dimension; 75.92% with the Working Together dimension; and 59.26% with the Student-Student Relationship dimension. Overall Engagement was high (3.98 ± 0.52) out of 5). Scores at the end of the program were similar to those obtained mid-program (p>0.05). Correlations: significant positive relationship between Intrinsic Motivation and the following variables: Knowledge [Pearson’s Correlation (r)=0.18; p=0.04], Performance Motivation (r=0.31; p<0.001), Engagement (r=0.64; p<0.001), Cognitive Presence (r=0.41, p<0.001), Social Presence (r=0.37; p<0.001) and Teaching Presence (r=0.39; p<0.001); as well as a significant negative relationship with Extrinsic Motivation (r=−0.24; p=0.01). | yes |
| Effectiveness of a blended learning intervention in cardiac physiotherapy. A randomized controlled trial | Marques-Sule et al. (2023b) | longitudinal single-blinded randomized trial with two arms of intervention | undergraduate physiotherapy students | 100 | studying the subject of cardiac physiotherapy as part of the Degree of Physiotherapy at the University of Valencia | previous cardiac physiotherapy training | hybrid | blended learning: asynchronous online learning modules, discussion forums, online material | both interventions lasted 2 months and included five face-to-face lessons and autonomous work using different methodologies according to the assigned group. Content and training for both groups was identical, and the only aspect that varied was the methodology. Paced asynchronous online learning modules and scheduled online activities with strategic face-to-face lectures were offered at set points during the semester. The students had autonomy and flexibility when accessing the online course content except for the intermittently scheduled face-to-face classes. Online resources for BLG: (1) LMS where the students were able to read breaking news, have access to the teaching guide, and solve any doubt through discussion forums; (2) online syllabus about cardiac physiotherapy consisting of five themes, including the same topics as the FLG; (3) online glossary comprised of the same terms as the glossary developed for the BLG; (4) online videos by international and national scientific societies, also aimed at improving the same aspects as reported in the FLG, such as knowledge regarding heart disease as well as reinforcing the concepts of professional deontology, equity, clinical status, and the experiences of vulnerable patients with heart disease; (5) several links to the websites of international and national scientific societies; (6) online activities with the same four questions per theme as the FLG were created, and personalized comments about the activities were also reported by the teachers in order to give feedback to the students; (7) the app Ariadna (Spanish Society of Cardiology) used to prevent cardiovascular risk and to look for defibrillators in the area; and (8) online tutorship through an online communication platform. | face to face learning: all information was provided on paper. Synchronous scheduled face-toface lectures (five in total) and scheduled activities offered at set points during the semester. | knowledge: MCQ; cross-curricular ethical and gender competencies: Higher Education, Transversal Skills and Gender Questionnaire; satisfaction: 4-point Likert scale; perceptions and evaluation of the intervention: 8-item Kavadella Perceptions Questionnaire; usability of the Moodle Virtual Classroom platform: System Usability Scale (SUS) | knowledge: BLG showed a significantly higher score than the FLG group (p=0.011; d=0.827) after the interventions. Satisfaction: BLG showed higher scores for increased motivation to prepare themselves before class (p=0.005; d=0.974), increased motivation and ability of thinking (p=0.005; d=0.987), and improved understanding of important and difficult topics (p=0.015; d=0.822) than the FLG. No significant differences between the groups for active class atmosphere, promoted teamwork spirit and ability, or developed clinical problem-solving skills (p>0.05). Perceptions and evaluation of the intervention: BLG presented with higher scores for course organization (p=0.017; d=0.828), educational material (p=0.001; d=1.236), easiness to understand (p=0.007; d=0.958), comprehensive coverage of the subject (p=0.001; d=1.218), and clarity of instructions (p=0.004; d=1.026) compared to the FLG. No significant differences between the groups in terms of perception and evaluation regarding course content, motivating study timetable, and the self-assessment test (p>0.05). Usability: SUS total score was 76.9 ± 15.2 out of 100 points, thus acceptable usability was reported. BL acceptance showed that the values of perceived easiness of use and usefulness were higher than 6 over 7 points, whilst attitude toward use and behavioral intention to use had values >5.5 over 7. | yes |
| E-learning for self-management support: introducing blended learning for graduate students – a cohort study | Munro et al. (2018) | cohort study | graduate entry medical program students + master of physiotherapy program students (all 2nd year) - report in the following fields only for the physiotherapy students | 183 in 3 groups (102 traditional, 52 blended, 29 e-learning) | NI | previously received formal self-managment system (SMS) training in their postgraduate courses | hybrid (1 group) and digital (1 group) | (1) blended approach (combining e-learning and face-to-face teaching) and (2) e-learning alone | training in the Flinders Chronic Condition Management Program: teaching a structured assessment approach, using processes and tools that explore self-management skills in partnership with patients, leading to the formulation of a self-management care plan. The material was presented to the students either face-to-face by an academic in a traditional 3h-lecture or via access to an online module. The module was assessed independently. Role playing session followed. Feedback session was conducted either in a 3h-lecture style format with the academic being present for the traditional and blended groups, or via videoconference with the academic for the e-learning group. This feedback session provided students with the opportunity to reconcile their knowledge of providing SMS. For their assessment, students utilized the Flinders model together with a patient. Students then had to submit the set of Flinders tools used in care planning along with a completed care plan for assessment. | traditional face-to-face delivery of SMS training | students’ competency in adherence to effective care plan processes: graded assessment (care plan + self-reflection on the Flinders Program process) | competences: the group that received a blended model of training had a significantly higher score for problem and goals (p<0.0001), care plan (p<0.0001), total score (p<0.0001), and engagement score (p<0.0001) compared to the traditional face-to-face group. There were no significant differences between the blended and the e-learning group across any of the other domains. | yes |
| eLearning in Physical Therapy: Lessons Learned From Transitioning a Professional Education Program to Full eLearning During the COVID-19 Pandemic | Ng et al. (2021) | cross-sectional qualitative study | undergraduate physiotherapy students enrolled in the 1st, 2nd and 3d year | 28 students in 7 focus groups | heterogeneity | NI | digital | eLearning: lectures delivered recorded or streamed with interactive features | Lectures and practical classes that were previously delivered face-to-face were shifted to fully online in the same scheduled time slots. Lectures were delivered through Echo360 video platform and videoconferencing. Both platforms allow recording, streaming, and interactive features. The latter was also used for practical classes to randomly distribute students into smaller groups. Group sizes varied from 3 to 6 students in each breakout group depending on the learning activity. Tutors facilitated a variety of learning activities, such as case-based learning, think-pair-share, and the use of workbooks with images and questions specifically developed for eLearning. Students were also encouraged to practice clinical skills on an individual basis in their household in front of their web camera to receive instant feedback from tutors or asynchronous feedback on video assignments students were asked to submit. Additional videos were created to supplement practical classes, such as chunked lectures (i.e., grouping of lectures into short, meaningful videos for online delivery) and additional clinical skills demonstrations that were previously only delivered face-to-face. Tutors joined all separate breakout groups at different times to discuss student queries and provide feedback on their activities. | NA | semistructured focus group interviews with 2-5 participants 4-5 weeks after transitioning to full eLearning; phenomenological interview framework: contextualization, apprehension of the changes, clarification of changes, barriers and enablers for eLearning | Three overarching themes were identified: (1) heightened negative feelings; (2) face-to-face learning is important; and (3) recommendations for eLearning | no |
| Remote‑online Case‑Based Learning: A Comparison of Remote‑online and Face‑to‑face, Case‑Based Learning ‑ A Randomized Controlled Trial | Nicklen et al. (2016) | mixed method randomized controlled trial | 3rd year undergraduate physiotherapy students | 38 (19 per group; 8 for the focus group) | NI | NI | digital | remote‑online case based learning (RO-CBL): case was presented via remote‑online access, in the form of web‑conferencing (users were able to utilize the online workspace much like a white board, and use a common screen to share notes, as well as view, upload, and share documents) | the CBL “case” was presented to students at the beginning of the academic week (Part 1). This gives context to the classes held throughout the week, during which the students develop the skills and knowledge to complete the case by the end of the academic week (Part 2). Students participated in two CBL sessions, Part 1 and Part 2, and both the control and intervention group ran simultaneously for approx. 90min. The CBL encouraged students to consider the role of the physiotherapist during the stages of pregnancy. | traditional face‑to‑face methods: paper based case scenario, access to textbokks and the internet | postintervention survey (MCQ + Likert Scale questions): learning and self‑assessed perception of learning, satisfaction, and participant demographics / 30min Focus Group interviews: overall reactions, ways to improve the RO‑CBL and applications to future learning | learning: median scores were comparable between groups (intervention: 9 [8–10], control: 10 [7–10]; p=0.61). Of the 15 examinable learning objectives, 8 were significantly different based on the Kruscal–Wallis rank test. All of these were in favor of the control. Only two had different median values compared to the intervention group. Perception + satisfaction: 16 participants (84%) disagreed with the statement “I enjoyed the method of CBL delivery” with 12 (63%) disagreeing with the statement “I felt I was able to achieve all objectives given the method of CBL delivery.”. Focus group key themes: risks to implementation, challenges to communication, and flexibility. | no |
| Development and evaluation of a 3D mobile application for learning manual therapy in the physiotherapy laboratory | Noguera et al. (2013) | preliminary study: cross-sectional evaluation; comparative study: crossover design | preliminary study: physiotherapy professors + 2nd-year undergraduate physiotherapy students; comparative study: 2nd-year undergraduate physiotherapy students | preliminary study: 14 professors + 70 students; comparative study: 76 | NI | NI | digital | mobile application for anatomical structures | preliminary study: mobile device was used in five practical sessions at the teaching laboratory as explained above. During each of these practical sessions manual therapy was taught in relation to five different anatomical areas: knee, ankle, pelvis, elbow and skull. Comparative study: 2x5h practical lessons: anatomy review of the body area to be palpated during the session. The review was followed by a description of different manipulative techniques and a practical demonstration performed by the professor. After the professor’s explanations, students had to practice the respective manipulation technique in pairs (one of them simulating a patient). Each pair of students was provided with a 4th generation iPod Touch device with pre-installed application. The mobile devices were placed on the stretchers next to the “simulated patients” and were configured to show the 3D model of the anatomy being manipulated by the student. They could freely adjust the rotation, zoom and pan of the 3D models according to their needs. (1) manual therapy was taught in relation to the knee and the ankle. For the knee the 2D view was used, whereas we employed the 3D view for the ankle. (2) the second session was about the pelvic zone. In this case, the 3D view was used. Groups changed between experimental/comparison in between the sessions. | traditional method (no mobile devices) | preliminary study: management of the device, user’s opinion of the device, application utilities, tool assessment, comparing 2D and 3D view modes: anonymous questionnaire (for the students: 20 limited response choice, 2 open ended / for the professors: 13 limited response choice, 2 open ended); comparative study: anatomical knowledge: posttest questionnaires after each session | preliminary study: all evaluations were above 4 (range 1–5) or above 9 (range 1–10). Both groups (professors+students) favored the proposal, but the evaluations of the students were again significantly higher. Comparative study: (1) significant differences were found between the two groups: mean difference between groups was 2.07 (95% CI: 1.66–2.47) in a range of 0–8. Considering that 100% corresponds to the eight questions answered correctly, this suggests a significant increase of over 25% of learning achievement for the experimental group. In this case (practical lesson about the knee and ankle) the proposed 3D m-learning tool was more effective than classical teaching methodology (2) significant difference was found between the two groups: mean difference between the groups was 0.7 (95% CI: 0.3–1.37) in a range of 0–8. This suggests a small increase of about 8% of learning achievement for the experimental group. In this case (practical lesson about the pelvic zone) the 3D m-learning tool had a better learning outcome than the traditional methods, but with less effect compared to the first practical lesson. | yes |
| Physiotherapy Students' Shaping of Digital Learning Practices: Characteristics, Opportunities and Challenges | Ødegaard et al. (2023) | qualitative study with individual in-depth interviews | physiotherapy students | 11 | NI | NI | digital | digital education practices | not further explained | NA | semi-structured individual in-depth interview: digital learning practices, how the students incorporate these practices into their studies, and their perspectives on the significance of digital education within their study programmes. | Two main themes and five sub-themes: (1) shaping of informal digital learning practices: (1.1) students’ confidence in their technological skills (1.2) combining different digital formats to increase learning (1.3) collaborative elements in digital work; (2) students’ ambiguity in positioning their digital learning practices: (2.1) reliability and pertinence of digital formats (2.2) challenges in aligning students’ digital learning practices with existing practice competence domains (2.3) limited linkages between current digital learning practices and clinical skills | yes |
| Evaluation of physiotherapy students’ attitudes and engagement with social networking and eLearning systems: implications for education and training | Pepera et al. (2022) | cross-sectional mixed-methods descriptive design | undergraduate physiotherapy students | 110 | NI | NI | digital | social networking sites (SNSs) and eLearning platform (eClass) | not further explained | NA | online survey multi-section questionnaire (34 questions; open-ended, MCQ, Likert-Scale questions): demographic characteristics, opinions in SNSs use, application of SNSs into education and the use of e-Learning platforms (eClass) | 99% of students use SNSs and all of them were aware of the existence of eClass in the Department of Physiotherapy and had made use of it. Overall SNS experience is generally high (64% more than 5 years). Use of SNS for entertainment and education purposes: 5%. 44% of students believe that SNSs can positively affect learning and education. A great majority (80%) of students reported that further training on SNSs can be highly effective in improving academic performance and learning. 70% of students supported that applying eClass to the education process is very helpful to construct and share knowledge. The majority of the students (97%) who did use their social networks as part of their studies used it as access to educational material whilst 68% for obtaining information on announcements and 36% stated accessible and instantaneous communication between university staff and students. | yes |
| The Physiotherapy eSkills Training Online resource improves performance of practical skills: a controlled trial | Preston et al. (2012) | non-randomized controlled trial | graduate-entry physiotherapy students | 59 (24: control group; 35: experimental group | first neurological physiotherapy unit of study in 2010 and 2011 | NI | digital | physiotherapy eSkills training online resource | EG: received access to 50 practical skills for management of stroke on the Physiotherapy eSkills Training Online resource over the last 5-week period of a 10-week teaching semester as well as during revision week, in addition to usual teaching. The resource incorporates 88 practical skills related to neurological physiotherapy. For each practical skill, the resource includes: (1) an on demand web-streamed high quality video-clip of therapist-patient simulation; (2) text describing the aim, rationale, equipment, key points, common errors and methods of progression; and (3) a downloadable PDF document incorporating the online text information and a still image of the video-clip. | usual teaching: 2 hours of lectures in a large group, and 4 hours of tutorials in small groups (≤ 16 students) per week over 10 weeks. Tutorials incorporated explicit teaching, case-based learning, problembased learning and live practical skill demonstration and student practice. | performance of practical skills: practical examination (25-point marking schema); usefulness of the Physiotherapy eSkills Training Online resource for learning practical skills (4 items): 10cm VAS | performance: EG scored 1.6 points out of 25 (95% CI −0.1 to 3.3) higher than the CG in the practical examination, 0.5 points out of 4 (95% CI 0 to 1.1) higher than the CG for the component ‘effectiveness of the practical skill’ and 0.6 points out of 4 (95% CI 0.1 – 1.1) higher for the component ‘rationale for the practical skill’. Usefulness: EG rated the Physiotherapy eSkills Training Online resource for: 1. improving their practical skills as 8.4 out of 10 (95% CI 8.0 to 8.7) 2. helping in examination preparation as 8.7 out of 10 (95% CI 8.3 to 9.1) 3. helping on neurological clinical practicum as 7.6 out of 10 (95% CI 7.0 to 8.2) 4. usefulness as a new graduate as 6.6 out of 10 (95% CI 5.8 to 7.4) | yes |
| Blended teaching versus traditional teaching for undergraduate physiotherapy students at the University of the Witwatersrand | Ravat et al. (2021) | cross-sectional study | 3d-year undergraduate physiotherapy students | 47 | NI | 3rd-year physiotherapy students who were repeating their year of study | hybrid | orthopaedics content using a blended teaching approach: revision quiz, online activities and face-to-face lectures | online activities consisted of videos sourced from the internet, podcasts, group case studies and online quizzes in lesson plans on the LMS; 6 online components, eight face-to-face lectures and the face-to-face debate task. At the end of the blended teaching orthopaedic teaching period, the students were taken to one of the academic hospitals, where they were orientated to the clinical setting, and they had the opportunity to assess and treat patients under supervision. | traditional face-to-face teaching (neuromusculoskeletal (NMS) and cardiopulmonary content (CP)): CP knowledge area consisted of 13 face-to-face lectures and three practical sessions. The NMS knowledge area consisted of two lectures and two practical sessions. At the end of the teaching period in both CP and NMS, and similar to orthopaedics, the students were taken to a clinical placement area, where they were orientated to the clinical area and assessed and treated patients under supervision. | performance of the students on all knowledge areas: knowledge test at the end of the teaching block, and a clinical performance mark was given at the end of the clinical placement; engagement and affect, knowledge and learning, and the blended teaching process: questionnaire (5-Point Likert Scale) | knowledge: significantly higher theoretical marks for orthopaedics were calculated compared to NMS and CP for both semesters with a large positive effect (average Cohen's d=4.44) for blended teaching on theoretical examination performance. No statistically significant difference between students’ orthopaedics clinical performance assessment in both semesters in the clinical summative assessment marks when compared with their performance in the NMS and CP areas (average Cohen’s d =–0.26). Engagement and affect: 74.4% felt that they were more engaged. 67.4% felt that the amount of interaction with other students increased, whilst 46.5% felt that interaction with the lecturer increased through the blended teaching approach. Whilst 32.6% of students felt overwhelmed by the resources in the course, only 16.3% felt that blended teaching made them more anxious. Knowledge and learning: students predominantly agreed or strongly agreed that the web resources were helpful, that a blended approach to teaching provided more opportunities to use and access information, that resources were easy to access, that they understood concepts better and that their understanding of the course material was improved. Blended teaching process: the majority of students indicated that using their devices for learning was useful (83.7%) and that they were able to use the technology and software needed to complete the course (79.1%). The students (83.7%) also believed that the online and face-to-face components of the course enhanced each other, and 86% agreed that they would take another blended teaching course if given the opportunity. 72% of students indicated that they would prefer a blended teaching approach. | yes |
| Flipping the classroom in physiotherapy education: experiences, opportunities and challenges | Røe et al. (2019a) | cross-over design | 2nd year physiotherapy students | 39 | NI | NI | hybrid | flipped classroom intervention: course on musculoskeletal disorders | practice period and an individual oral exam, then three weeks of traditional lectures, then three-week period of flipped classroom education. Flipped classroom: pre-classroom digital learning resources, and in-class collaborative learning activities. Digital learning resources (22 digital lectures/5 hours in total): YouTube videos, podcasts and blog posts. Digital lectures were made available for the students at least one week before the in-class activities. In-class learning activities consisted of 6 full-day seminars where the students worked on assignments. Each seminar focused on a theme (i.e. persistent pain and evidence-based physiotherapy). A leaflet, with exercises for each seminar, was distributed to the students. The exercises were theoretical, with different levels of difficulty. Typically, the students would start with exercises requiring a brief answer, and end with more complex assignments, typically patient cases (group assignement, 5h). 45min plenary discussion afterwards | NA | students’ opinions: survey; ratings of overall self-perceived learning outcomes: 10-point NRS | students opinions: autonomous group-work, unlimited access to digital material; frequent negative factors: lack of joint summary at the end of the seminars, long duration of seminars and little variation in learning activities. Perception: significant difference in the scores for student satisfaction of the flipped classroom intervention (M=6.85, SD=1.39) and traditional lectures (M=5.62, SD=1.33); t(38)= –4.459, p<0.001. | yes |
| Learning with technology in physiotherapy education: design, implementation and evaluation of a flipped classroom teaching approach | Røe et al. (2019b) | historically controlled, prospective, cohort study | 2nd year physiotherapy students | 54 | NI | NI | hybrid | flipped classroom intervention: course on musculoskeletal disorders: pre-recorded video lectures, YouTube-videos, podcasts and an e-learning course | 8 weeks course (7 full day seminars). Pre-class learning resources consisted of about 12h of pre-recorded video lectures, YouTube-videos, podcasts and an e-learning course. In addition, several key scientific papers, were included. The digital learning resources were organised in seven themes and made available for the students a week in advance of the course. All seminars had a similar structure, starting with a plenary session of about 45 min (possibility to ask questions). The plenary session was followed by 5h, where the students were working in groups, solving assignments. The end of the seminars was devoted to student presentations where two groups exchanged and discussed answers of the assignments. | cohorts from 2013-2016 | student performance/learning outcome: course exams; student perceptions: survey (6 open-ended questions) | course exams: the share of students with excellent, very good and good performances showed a higher rate by more than 10% relative to any previous year. Satisfactory, sufficient and fail performances showed a lower rate by more than 10%. Survey: 29 students reported that they preferred the flipped classroom approach. 20 students reported that they preferred a more structured classroom. most frequently reported factors that represent students’ view from the seminars were interaction with peers and educators, and the flexibility associated with digital learning resources. | yes |
| Online teaching in physiotherapy educationduring COVID-19 pandemic in Italy: aretrospective case-control study onstudents’ satisfaction and performance | Rossettini et al. (2021) | retrospective case-control study | entry-level physiotherapy students | 158 (online group: 46, face-to-face conventional lessons: 112) | NI | NI | digital | online lectures + asynchronous online lectures + eMail/Chat communication with provision of supplementary online ressources | course: advanced methodologies in musculoskeletal physiotherapy. 3 main topics: clinical reasoning, analysis of pain mechanisms and evidence-based physiotherapy practice. 50-60h workload, 24h face-to-face lectures converted to online. | face-to-face courses of the previous 5 academic years | students’ satisfaction: standardized national-established 12-item questionnaire (5-point Likert Scale); performance: oral exam | performance: significant difference in the mean performances online teaching vs. face-to-face teaching in favor for the online teaching group: Wilcoxon rank sum test W=1665, p<0.001. Satisfaction: no difference between the groups in the perceived satisfaction of the course (Kruskal-Wallis Χ2 =3, df=1, p=0.08). | yes |
| Competence Development in an Undergraduate Physiotherapy Internship Program during the COVID-19 Pandemic: A Blended Learning Approach | Ruiz-Ruiz et al. (2023) | analytical retrospective study | physiotherapy students | 300 (150: experimental group, 150: control group) | NI | NI | hybrid | blended teaching methods, including face-to-face learning: 50% attendance at the clinical practice center and 50% completion of asynchronous online complementary training | course: clinical stay II (3rd-year subject consisting of 200 clinical presential hours, prerequisite for practicum). 6 asynchronous visualization videos focused on the work of clinical reasoning, communication, decision-making, conducting patient anamnesis, and integration of practical and theoretical knowledge by applying it to specific clinical cases and providing effective and comprehensive care. The tutor communicated with the students through a virtual platform explaining the activity and the evaluation mode, as well as the evaluation criteria and objectives. | face-to-face learning | academic marks: Clinical Stay II - 40% out of an 20-MC-Q; Internship - Clinical Practice: 70%; Clinical History: 24%; Reflective Diary: 6% | academic marks: there were no significant differences in the median of the evaluations between the cohorts, either in Clinical Stay II or Practicum; CG: no significant difference (p>0.05) was found in the median of the evaluations between subjects of the two study courses, even though there was a tendency for the Clinical Practice and Clinical History scores to be higher in the Practicum in contrast to Clinical Stay II. In the EG, a significant difference was found between the median of the Clinical Stay II and the Practicum total scores (p<0.001). The median of the total score of the Practicum was slightly lower than the Clinical Stay II total score (8.34 vs. 8.69) | no |
| Opinions of Students Studying in Associate Degree Health-Related Departments Requiring Professional Practice and Skills toward Web-Based Distance Education during the Pandemic: A Cross-Sectional-Descriptive Study | Sari et al. (2022) | cross sectional-descriptive study | home care, elderly care, physiotherapy, and first and emergency aid students | 264 (74 physiotherapy students) | NI | NI | digital | online learning, web-based distance education | not further explained | NA | online questionnaire (48 items): students’ views on the materials used for distance education, quality of internet, quality of the education provided, difficulty in doing homework, the sufficiency of exam evaluation, and fear of working after graduation | "web-based distance education is the right method of education": 47% of the physiotherapy students answered no, 27% answered yes | no |
| Analysis of different gamification-based teaching resources for physiotherapy students: a comparative study | Sandoval-Hernández et al. (2023) | comparative study | 4th-year physiotherapy students | 33 | (1) being over 18 years old, (2) being enrolled in the elective subject of First Aid from Physiotherapy. | (1) positive COVID-19 test, which made it impossible to participate in the classes where each of the gamification resources were developed (Kahoot!, Physiotherapy Party and Escape Room), (2) cancelation of enrollment in the subject during the semester | digital | game-based learning: three gamification resources: Kahoot!, Physiotherapy Party and Escape Room | course: “First Aid from Physiotherapy”: consists in the acquisition of practical skills and theoretical content, where students are organized in groups of 15–20 students to develop practical content. Introduction of generalities of First Aid from Physiotherapy, basic cardiopulmonary resuscitation, airway obstruction, trauma injuries, hemorrhage, skin aggression, emergency delivery, alterations of consciousness, aggression by physical or environmental agents and intoxication. **Kahoot!** allows teachers to create four different types of games, only quizzes and true-false questions were used, where participants compete against each other. A Kahoot! was performed at the end of each topic of the theoretical content. The total time spent on this resource was 1h in all. **Physiotherapy Party:** objective of this gamification resource is for the participants to win each of the different challenges in mimicry, questions, forbidden words and drawings about the contents of the subject. The cards were made by the students during the course as they completed the course topics. The total time spent on this resource was 1h. **Escape Room:** participants are introduced to a scenario where they must solve puzzles and use clues to complete the activity and thus escape from the room within a certain amount of time. To solve the clinical case, they had to communicate by walkie talky and complete all the tests. Among the tests, they had to decipher a hidden message with ultraviolet light, open locks and safes and correctly carry out the first-aid protocols learned in the subject. The total time spent on this resource was 1h. | For the comparisons between the three gamification tools | gaming experience: GAMEX gaming experience scale | experience: in the enjoyment dimension, differences exist between Physiotherapy Party (p=0.05) and Escape Room (p=0.025) with respect to Kahoot!, as well as in creative thinking (p≤0.001). On the other hand, in the absorption dimension, the differences exist between Escape Room and Kahoot! (p=0.001). Lastly, in the dominance dimension, there were differences between Escape Room and Kahoot! (p=0.037). | not clear, as it was not compared to traditional learning methods |
| Learning promotion of physiotherapy in neurological diseases: Design and application of a virtual reality‑based game | Shahmoradi et al. (2020) | cross-sectional study | undergraduate physiotherapy students | 31 | NI | NI | digital | application of VR games in education | course: Physiotherapy in Neurological Diseases. Brief description of VR and its applications was given. The common exercises for physiotherapy of neurological patients in the form of five games (spacecraft, falling snow, butterfly, tubes, and sorting of home) were taught to students. Each game could generate movements in the patient’s upper limb. During the training, the professor answered the students’ questions. | NA | student’s perception of the learning: adapted Dundee Ready Educational Environment Measure (DREEM) questionnaire (10 items, 5-Point-Likert Scale); facilitating ability of VR + student satisfaction: self-developed questionnaire (8 items, 10-Point-Likert Scale) | DREEM: the mean (standard deviation) of the total scores of the first nine questions related to the students’ perception of learning was 2.95 (0.70) with a median score of 3.11. Question 10 was reversed and its mean (standard deviation) and median scores were 2.2 (0.71) and 2, respectively. Facilitating level of the game in education: the mean (standard deviation) and median of the three questions in this section were 8.04 (2.18) and 8.66, respectively. Student’s satisfaction in using the game in education: the mean (standard deviation) and median of the first four questions in this section were 8.27 (2.07) and 9, respectively. Question 5 in the second part of the questionnaire was reversed. Thus, the mean (standard deviation) and median of the 10 were 1.79 and 1, respectively. | yes |
| E-learning communication skills training for physiotherapy students: A two phased sequential mixed methods study | Soundy et al. (2021) | two phase sequential explanatory mixed methods study | undergraduate + postgraduate physiotherapy students | 25 (12 in the experimental group; 13 in the control group) | NI | currently part of other communication or psychologybased research | digital | asynchronous seminars: pre-recorded e-learning seminar using the model of emotions, adaptation and hope (MEAH) | students were randomly allocated to a single 1h-lecture of a novel narrative-based intervention, supported by the MEAH or allocated to a control group. The MEAH training had 45 slides directed by 5 core elements; (1) a specific focus on non-judgemental and non-directive aspects of communication, (2) the light bulb analogy of LED (Listen, Explore, Direct) is introduced to enhance communication. Continuous examples are given that use LED within highly challenging interactions (3) an introduction of illness narrative master plots (common stories of illness) and the hidden psychological meaning within the plots are given (4) traditional understanding of psychological adaptation and goal setting is problematised and compared against the MEAH (5) the MEAH is identified as a psychological map of illness narrative master plots. The implications of this for assessment and support of patients is provided. | standard pre-recorded motivational interviewing session | empathy: Interpersonal Reactivity Index (IRI); stigmatising attitudes: Mental Illness Clinicians’ Attitudes Scale (MICA); stigmatising attitudes and intentions of behavior: Open Minds Scale for health care providers; self efficacy: General Self Efficacy Scale (GSE); commucation skills: Froehlich Communication Survey; survey (open ended questions): Qualitative process evaluation; perception and experiences of the intervention, past training, and future application: semi-structured interview | questionnaires: there were no significant between group differences for all outcome measurements. There were significant differences for time for the personal distress subscale from baseline to 6-weeks for the intervention group, Froehlich Scale at baseline to post intervention for the intervention group and the Froehlich Scale and the GSE for the control group from baseline to 6-week. Qualitative process evaluation: all participants said the e-learning intervention had impacted on them as a therapist and personally and 11 (11/12, 91%) said that future courses should include the course. The most frequently identified impact as a therapist was the perceived ability now to manage, handle and respond to people who are facing challenging situations associated with their diagnosis and symptoms (n = 8/12, 67%). At an individual level, the most common response was a greater understanding of the different ways people may cope with problems or difficult situations (n = 7/12, 57%). 8 students (8/12, 67%) wanted more content related to the intervention. Semi-structured interviews: 5 themes were identified; (1) confidence, (2) factors which influenced confidence, (3) empowerment of the patient, (4) factors which influenced empowerment of patients and (5) considerations around implementation of the approach. | yes |
| A comparison of physiotherapystudents’ perception about blendedlearning with online learning duringCOVID‑19 pandemic: A mixed methodof study | Swaminathan et al. (2022) | mixed‑method study | graduates and postgraduate students | 68 | NI | NI | hybrid | blended learning courses with synchronous online components | 2 courses: Evidence‑Based Physiotherapy Practice (EBP) + Transferring Research in to Practice. Students had access to course contents through the dedicated LMS of the institute. Synchronous online components included video tutorials, external links, reading materials, automated quiz, discussion forums and assignments. Face‑to‑face sessions included small group discussions, tutorials, digital quizzes, interactive lectures, and group presentations. Learners received individualized feedback through online and face‑to‑face sessions during the course. | traditional courses delivered via online learning (OL) | students perception about BL compared to OL: questionnaire; perception and experiences: focus group discussion; learning outcomes: continous + summative assessments | questionnaire: most respondents reported that the course enhanced their knowledge and skills in EBP, and they were satisfied with course contents, delivery, and assessment methods adopted. Most participants (93%) recommended BL compared to OL. Participants perceived that BL provided a better opportunity to interact with their peers and faculty members (91%). According to the respondents, more in‑depth learning happened in BL (88%) when compared to online learning alone (12%). Focus group interviews: 3 main themes from 7 categories: (1) learning experiences: (1.1) access to learning materials, (1.2) active learning, (1.3) understanding of the subject; (2) collaborative learning: (2.1) peer‑to‑peer collaboration, (2.2) learner‑teacher collaboration; (3) perceived barriers: (3.1) quality of content, (3.2) availability of gadgets for online learning and internet. | yes |
| Interactive virtual scenarios as a technological resource to improve musculoskeletal clinical reasoning skills of undergraduate physiotherapy students | Torres et al. (2022) | quantitative quasi-experimental study | 8th semester undergraduate physiotherapy students | 92 | NI | NI | hybrid | blended training with interactive virtual scenarios (storylines) for the development of clinical reasoning skills; using videos, audios, images, and texts | musculoskeletal course. Each case (virtual scenario) represented a characteristic pattern of clinical presentation. In each storyline, the student had to make decisions (tree type) about the patient. These decisions allowed them to move through the case until completing the assessment and reaching the final clinical reasoning decision. Three parts: (1) Interview: students had to choose questions that were relevant according to the presented case, (2) Physical Assessment: students had to choose appropriate clinical tests for each virtual patient, (3) Interpretation of the results. | NA | performance of clinical reasoning: error percentage (rate of incorrect answers during interview and physical assessment parts); clinical pattern recognition: online survey; ability to recognize the correct clinical pattern: MCQ ; perception of difficulty: online survey (5-Point-Likert Scale); learning satisfaction: validated online survey (14 items, 5-point-Likert Scale) | clinical reasoning skills: the clinical pattern in the written cases before the intervention fluctuated between 52.2% (95% CI 41.9–62.5) for the first case and 68.9% for the third case (95% CI 59.3–78.5). The error percentage decreased after the intervention, fluctuating between 7.6% (95% CI 2.2–13.0) for the first and second case, and 17.4% (95% CI 9.7–25.1) for the third case. All comparisons in error percentage between the baseline and final assessment were statistically significant (p<0.001). There are significant differences in the obtained error proportions, as the intervention progressed (p<0.01). Pattern recognition correctness: for each error percentage point, the probability to correctly recognize the clinical pattern decreased by 11% (OR=0.89; 95% CI 0.85–0.93). Perception of difficulty: the first storyline stands out, which resulted statistically different from the one obtained in the eighth storyline (p<0.001). Learning satisfaction: large number of students indicated to agree or strongly agree with the assertions: “Storyline organization facilitated my learning” (97.9%), “The use of storylines promotes selflearning” (96.8%), “Storylines helped me improve my pattern recognition” (97.9%), and “Storylines helped me improve my clinical reasoning” (96.7%). More significant dispersion in the answers of the following assertions: “The use of storylines helped me improve my confidence before beginning my professional placement” with a 73.2% agree or strongly agree, and “The storyline format has clear instructions” with a 70.9%. | yes |
| Learning effectiveness of 360° video: experiences from a controlled experiment in healthcare education | Ulrich et al. (2021) | randomized controlled trial | physiotherapy students | 83 | start of education a few days prior to the experiment | NI | digital | 360° video shown on a virtual reality head-mounted display | practical task of performing correct supine positioning. The lesson instructed the students on (1) the importance of supine positioning, (2) abbreviations used, (3) correct deployment of equipment, (4) correct adjustment and preparation of the medical bed, (5) correct resting position of the neck and knees, (6) how to create a loose-pack position of the knee in a supine positioning, and (7) what the patient will do when he/she is not relaxed. | (1) regular video shown on a laptop; (2) traditional teaching absent of technology use (lesson from instructor) | academic performance: pre- and posttreatment tests; perceived user satisfaction + perception of learning climate: 13 Likert-scale items | academic performance: t-test demonstrated that the EG obtained a mean increase of 4.259 in the test scores, with 95% CI [3.832,4686], t(80)=19.875, p<0.000. ANOVA identified a small variance (not significant) between the three groups (F(2,81)=1.512, p=0.227), meaning that all three groups were equally effective. Learning satisfaction: significant difference within the composite for internal perspectives, χ2(2)=8.636, p=0.013. The composite for external perspectives yielded a significant result between the three treatment groups, χ2(2)=10.938, p=0.004. 360° video (mean rank=22.79) to be ranked lower than traditional teaching (mean rank=32.58), U=232, z=−2.688, p=0.007. Internal perspectives: regular video (mean rank=22.44) was outperformed by traditional teaching (mean rank=31.73), U=228, z=−2.598, p=0.009. No significant difference was found between 360° video (mean rank=28.50) and regular video (mean rank=27.48), U=364, z=55, p=0.799. External perspectives: 360° video (mean rank=23.68) was outperformed by traditional teaching (mean rank=31.62), U=257, z=−2.110, p=0.035. Likewise, regular video (mean rank=21.04) was ranked lower than traditional teaching (mean rank=33.19), U=190, z=−3.169, p=0.002. No significant differences between 360° video (mean rank=31.29) and regular video (mean rank=24.59), U=286, z=−1.611, p=0.107. Perception of learning climate: significant difference in the composite for communication, χ2(2)=38.780, p<0.000. The composite for emotions also contained a significant difference between the three groups, χ2(2)=18.221, p<0.000. In the student’s perception of communication in the learning climate, 360° video (mean rank=16.50) was outperformed by traditional teaching (mean rank=39.35), U=56, z=−5.364, p<0.000. Likewise, regular video (mean rank=16.00) was outperformed by traditional teaching (mean rank=38.42), U=54, z=−5.336, p<0.000. However, no significant differences were found between 360° video (mean rank=28.36) and regular video (mean rank=27.63), U=368, z=78, p=0.859. Regarding the student’s emotions about the learning environment, there was no significant difference between 360° video (mean rank=25.96) and traditional teaching (mean rank=29.15), U=321, z=53, p=0.452. However, 360° video (mean rank=35.43) outperformed regular video (mean rank=20.30), U=170, z=−3.507, p<0.000. Likewise, regular video (mean rank=19.15) was outperformed by traditional teaching (mean rank=35.15), U=139, z=−3.782, p<0.000. | no |
| Remote, asynchronous training and feedback enables development of neurodynamic skills in physiotherapy students | Villagrán et al. (2023) | longitudinal repeated measures study design | 3rd-year undergraduate physiotherapy students | 60 | NI | (1) did not complete the course within the standard timeframe (2) enrolled in the course for a second time | hybrid | remote training with asynchronous feedback for the development of neurodynamic skills | course: musculoskeletal physiotherapy assessment. 4 sessions of remote, asynchronous training of neurodynamic assessment tests. They practiced the Upper Limb Tension Test 1, Upper Limb Tension Test 2B, and the Upper Limb Tension Test 3 in each session of the remote training across 5 weeks. This strategy was based on the C1DO1 (“see one, do one”) platform, that engages students in sequential training and allows instructors to provide multimodal feedback (i.e., written, drawings, audio, or video) on students’ self-recordings. (1) on the platform, the student watched tutorial videos showing how to perform each technique step by step safely on a simulated patient/model, (2) following the tutorial video and with the help of a relative or friend posing as a patient/model, the student recorded a video performing the three techniques, consecutively, and uploaded their video onto the C1DO1 platform, (3) the instructor assessed the performance using a rubric, and provided multimodal feedback through the same platform, (4) the student received feedback and formative assessment via the rubric, and continued practicing the neurodynamic techniques until they felt ready to rerecord their performance and start the cycle again. This four-step process was repeated across four sessions with the same three neurodynamic techniques. Between each session, students had 5 workdays to review the assessment rubric and feedback from the previous session and rerecord the 3 techniques in their new video. | NA | experiences and perceptions: online survey; student performance: purpose-designed observation checklist | student performance: progressive improvement shown by the increase in the percentage of students achieving the target standard. Differences in the performance in at least one of the videos compared to the others (F(3,177)=44.53, p<0.001). Significant differences between Video 1 and 2 (t(59)=-6.67; p<0.01), between Videos 1 and 3 (t(59)=-8.54; p<0.001), between Videos 1 and 4 (t(59)=-9.18; p<0.001), and between Videos 2 and 4 (t(59)=-4.32); p<0.05). On the other hand, the difference between Videos 2 and 3 was not significant (t(59)=-2.27; p=0.36), and likewise the difference between Videos 3 and 4 was not significant (t(59)=-2.07; p=0.56). These results indicate that after session 2 (i.e., Video 2) students do not display a statistically significant difference in performance. For the rubric, there were differences in the performance in at least one of the videos compared to the others (F(3,177)=27.92, p<0.001). The post-hoc contrast for the rubric reported significant differences between Videos 1 and 2 (t(59)=-4.25; p<0.001), between Videos 1 and 3 (t(59)=-6.58; p<0.001), between Videos 1 and 4 (t(59)=-7.08; p<0.001), between Videos 2 and 3 (t(59)=-3.45; p<0.05) and between Videos 2 and 4 (t(59)=-4.39; p<0.001). On the other hand, the difference between Videos 3 and 4 was not significant (t(59)=-1.16; p=1). These results indicate that after session 3 (i.e., Video 3) students do not display a statistically significant difference in performance as indicated on the rubric. Student's perception: most participants agreed or fully agreed that the C1DO1 platform was easy to use (95%) and that the instructions were clear (91.7%). Regarding the training, 65% of the participants reported that they agreed or fully agreed that it allowed them to acquire practical skills, and 88.4% agreed or fully agreed that the feedback helped them to improve across sessions. Concerning confidence, the biggest percentage of participants moved from a perception of “insecurity” (43.3%) in their performance the first time to a perception of “confidence” (41.7%) or “high confidence” (33.3%) in their performance of the techniques after the training. In addition, the 48.3% of the participants stated that they felt prepared to perform the techniques on people with real conditions after completing the training. | yes |
| Satisfaction Level and Performance of Physiotherapy Students in the Knowledge of Musculoskeletal Disorders through Nearpod: Preliminary Reports | Vinolo-Gil et al. (2022) | intervention study | 3rd year physiotherapy students | 50 | NI | NI | hybrid | Nearpod: educational application based on a web-based learning tool. Can be used in conjunction with video conferencing tools to effectively engage students in a synchronous online classroom. Allows real-time quizzes, slides, videos, drawings, surveys, open-ended questions, web content, and various activities to be presented through an interactive presentation created and controlled by teachers. It can also be used as a formative assessment tool, as it is possible to obtain immediate feedback from students, or as an interactive tool, as students can participate in the content of the presentations through their answers, comments, or questions presented | course: Physiotherapy in Clinical Specialties II (musculoskeletal disorders). This project was carried out during 2h of theory. In one of the hours, the CG was with a subject teacher conducting a discussion on a topic unrelated to musculoskeletal disorders, while one of the project teachers was in another classroom with the EG using Nearpod. In the next hour, the CG went on to teach the musculoskeletal disorders class in the traditional way with the project teacher, and the EG went to the second classroom to conduct the discussion with the other subject teacher. | power point presentation | students’ satisfaction with the applied learning method: questionnaire of student’s satisfaction + 13-questions satisfaction survey; knowledge: posttest | satisfaction survey: a total of 83.33% thought that there were positive changes in terms of their dynamism and interactivity with this type of presentation compared to the traditional method, and 97.62% considered it effective in terms of attention gain and the use of this type of methodology had the possibility of increasing interest in other subjects. In terms of the perception of the students related to the level of interest that this teaching method provoked in them, they found it interesting (42.86%) or very interesting (54.76%). Approximately 88% thought that the time invested was adequate in relation to the level of knowledge acquired, and 97.62% considered that what they had learned with this type of practice had helped them to understand the contents of the subject. *Most repeated advantages:* dynamic, enjoyable, and interesting, increased attention, avoided boredom, interactive, entertaining, fun, innovative and helped to maintain concentration. *Drawbacks:* you had to be very attentive, some videos became monotonous, small technical problems with the internet, little time to answer the questions, could slow down the dynamics of the class. CSD: *Utility-Subscale* - in all 3 items, the results are higher than 7. All these data indicate that the surveyed students consider the application of Nearpod useful. *Organisation and resources-Subscale* - most of the answers are higher than 7. Both the resources and the time spent were adequate for the Nearpod activity. *Methodology-Subscale -* the average data obtained in these two questions exceeds an average of 8 points. The students consider that this type of didactic presentation is suitable to be used in their learning and assessment. Academic performance: the group or treatment does not have any influence, as its significance is 0.075 > 0.05 | yes: satisfaction, no: academic performance |
| Implementing a Virtual Care Clinical Placement for a Physiotherapy Student in a Hospital-Based Chronic Pain Programme | Vrzic et al. (2022) | case report | (1) entry-level master physiotherapy students (in 4th clinical placement), (2) clinical instructor (CI) in the chronic pain programme who supervised the student, (3) academic coordinator of clinical education (ACCE) in the MScPT programme who coordinated the placement | 3 | NI | NI | hybrid | part-time virtual care clinical placement in a hospital-based chronic pain programme | The placement consisted of 40 hours of supervised clinical education over 4 weeks (10h/wk). Clinical care was delivered virtually by telephone and through an interactive online audio-visual communication platform. While on clinical placement, the physiotherapy student maintained a caseload of five patients with chronic pain, providing initial assessments and follow-up care. At the end of each day, the student and CI met in person for 15–30 minutes to debrief and discuss each patient interaction. During these debriefing sessions, the student asked questions, and the CI provided feedback and direction on patient care. | NA | student’s performance: learning goals + Canadian Physiotherapy Assessment of Clinical Performance at mid-term (2 weeks) and end of term (4 weeks); reflections on the strengths of, challenges to, and future recommendations for the virtual care clinical placement: notes | *Results only from student's perspective:* student's performance: by the end of the clinical placement, the physiotherapy student had achieved their learning goals. Strengths: it fostered strong independence and communication skills, virtual care aligned well with this practice setting (e.g., patient education, self-management support, physical activity and exercise prescription). Challenges: navigating supervision when providing virtual care, providing and receiving immediate feedback. Recommendations for future clinical placements: future physiotherapy students who provide virtual care on their clinical placements should seek out opportunities to advance their own knowledge of, and comfort with, providing virtual care. | yes |
| Flipped classroom versus traditional teaching methods within musculoskeletal physical therapy: a case report | Wassinger et al. (2022) | case report | physiotherapy students | 29 | NI | NI | hybrid | Flipped classroom: online videos for all aspects of content delivery through condensed not longer than 1h streaming lectures. Online videos also included demonstrations of the psychomotor skills for the class meeting. Flipped class began with an individual readiness assessment test or quiz, followed by small group activities centered around developing application skills from the foundational knowledge used in the streaming lectures. They were used to promote watching the video lectures and to determine if students understood the main concepts. The small group learning activities employed written case reports, psychomotor skill development, clinical reasoning, red flag recognition, etc. At the end of the in-class meeting time, all students were brought together to review the inclass activities through student presentations and summaries by the instructors. | musculoskeletal course, team taught by 3 experienced faculty members over a 7-week summer term. Classes met 3 days per week for 3.5 hours per class session. The flipped classroom model was to be used by one faculty member teaching content of the cervical spine and elbow. TMJ, shoulder and wrist/ hand were taught by the other faculty members using traditional models with class lecture and lab components. All content, regardless of teaching method, was presented sequentially with review of anatomy and biomechanics, followed by presentation of common clinical conditions, with assessment and treatment considerations primarily patient education, manual therapy, and therapeutic exercise described and practiced last. | traditional teaching methods: in-class lecture delivery using power point presentations embedded with reflective questions, case examples, large group discussion and question/answer time. Lab time focused on psychomotor skill demonstration of assessment and treatment techniques presented in-class to the whole group followed by directed practice in groups of two or three students with instructor feedback. There were no required pre-class activities although students were encouraged to review associated textbook chapters in preparation for class. | student feedback regarding the teaching methods used and for future course planning: online survey; examination scores (compared to the previous cohort): MCQ | exam scores: outcomes on the examinations indicate little difference (<4%) for exam scores excluding the cervical spine exam which was taught in the flipped model and improved by >11%. Student preferences for teaching methods: students preferred the flipped classroom for all categories except desire to learn and preparation for course exams which were similar between flipped classroom teaching methods and both equally beneficial. Students also reported a lack of preparation for traditional lecture with 2/28 (7%) reviewing the chapter content prior to class, whereby 100% of students reported watching videos prior to class. Additionally, 86% of students reported relying on the lecture handouts more than the textbook for learning. 97% (23/29 students) described re-watching the videos after class for review or study. When asked outright 28/29 (97%) students preferred the flipped teaching method. | yes |
| A video-based learning activity is effective for preparing physiotherapy students for practical examinations | Weeks & Horan (2013) | qualitative and quantitatve data collection | 1st-year physiotherapy grauduate entrystudents | 62 | NI | NI | digital | video-based learning activity | course: viva preperation. Two clinical cases were constructed – one cardiorespiratory-based and the other neurological-based. Example viva questions were developed for each. A new-graduate physiotherapist was filmed performing both viva scenarios in examination format, led and assessed by an experienced examiner. For each scenario, the new-graduate was given a small amount of coaching so she could perform one at a level that could be considered good-excellent and another at a less satisfactory level to produce four individual video-based viva examples (each approximately 20 minutes). To maintain assessment standards, each video example was assessed by a consensus panel of experienced examiners including one academic staff member and one external clinician. A 2h workshop was developed to incorporate and present the video examples and to engage students in preparation for their viva examination. Held almost three weeks before the viva, it served as a method of actively communicating assessment standards using multiple exemplars. | previous year's cohort: identical to the current year in terms of planned content, number of practical class hours, and allocated clinical skills practice time. No video-based preparation workshop and associated web-based materials. | student perceptions around the use of videos: survey (5-Point-Likert Scale); student satisfaction: 8-item survey; example performances: student marks; viva performance: viva grades; perceptions of the new approach: focus group interviews | initial attitudes to video resources: students were very supportive (98% positive) of the use of video resources to aide their learning. Video demonstrations would be beneficial to support their viva preparation (95% positive), rating such demonstrations would be beneficial (83% positive), video examples placed on the course website would be beneficial to their learning (98% positive). Satisfaction of the video-based learning activity: students were very satisfied (93% positive) with the new video-based viva preparation session. Students felt the video resources were effective to support their learning and that rating the examples of was an effective learning activity (98% and 96% positive). Viewing the video examples was seen to be beneficial to support their viva preparation (98% positive) and rating the video examples improved their familiarity with the marking criteria and learning objectives (93% positive). Students suggested that the video examples improved their confidence in the viva (95% positive). Although 73% of responses were positive when asked about access to the video resources on the course website, 20% of responses were neutral, and 7% of responses were negative. Overall, students enjoyed the viva preparation session (93% positive) and felt that it should be employed in future courses (98% positive). Focus groups: the following sub-themes were identified from the two main themes: Supportive – (1) Accessible; (2) Understanding; (3) Reduced anxiety; (4) Enjoyable; (5) Good timing; and Critical – (6) Predictable. Performance and agreement: viva marks (out of 100) for students in the current cohort (n=62) were significantly better than for students in the previous cohort (n=50) (mean 81.6 SD 8.7 vs. mean 78.1 SD 9.0, p=0.01). Student awarded marks for the video examples were significantly lower than those awarded by the panel of examiners for both good (mean 90.4 SD 5.5 vs. 94.0, p<0.01) and poor (mean 38.2 SD 6.6 vs. 41.0, p<0.01) cardiorespiratory cases and the good neurological case (mean 87.0 SD 6.3 vs. 90.0, p<0.01), but were no different for the poor neurological case (mean 40.3 SD 6.8 vs. 41.0, p=0.42). | yes |
| Assessing the Educational Environment of a Flipped Physical Therapy Course: Utilization of the Dundee Ready Education Environment Measure (DREEM) | Willis et al. (2018) | quasi-experimental posttest-only design | first-year doctorate of physical therapy students | 117 (58 in EG; 59 in CG) | NI | NI | hybrid | flipped classroom model (FCM) | to implement the FCM, a new course instructor created 24 original online videos for students to view prior to in-person laboratory sessions. Each video averaged 12min in length. Students were required to view 2-3 videos per week prior to their laboratory session. Videos were accessed from the students’ online education management system of their university. The FCM laboratory sessions were 60min in length. 4 teaching assistants were employed to assist in content delivery and lead students through stations. The in-person laboratory sessions were completed in 6x1h labs of 10 students each, with 4 teaching assistants, which resulted in a student to instructor ratio of 2.5 to 1. | traditional teaching | Dundee Ready Education Environment Measure (DREEM): educational environment (EE) with the domains: students’ perceptions of learning (12 items), students’ perception of teachers (11 items), students’ academic self-perception (8 items), students’ perception of atmosphere (12 items), and students’ social self-perception (7 items); knowledge: cumulative grade point average | DREEM: the FCM demonstrated a mean global score of 168.2 ± 13.3, demonstrating an excellent EE. Mean DREEM domain scores for students’ perception of learning (41.3 ± 3.9), perception of teachers (39.6 ± 2.9), academic self-perception (25.1 ± 2.5), perception of atmosphere (40.1 ± 4.1), and social self-perception (22.1 ± 2.9) all fell into the highest rated subscale category. 26 items (52%) were identified as particularly strong areas (means scores of ≥ 3.5) and 5 items (10%) were identified as areas that could be improved (mean scores between 2 and 3) while no individual items required particular attention or concern (mean score ≤ 2.0). Knowledge: no significant differences were seen in cumulative course GPA between the flipped classroom model (3.74 ± 0.44) and the previous traditional teaching model (3.71 ± 0.46). | yes: students perception, no: knowledge |
| Active digital pedagogies as a substitute  for clinical placement during the COVID-19  pandemic: the case of physiotherapy education | Wojniusz et al. (2022) | result of a qualitative thematic analysis of the students’ essay assignments | 3rd and final year physiotherapy students | 43 | NI | NI | digital | e learning (video conferences) | Teaching activities were conducted digitally using video conferences. All students completed an online form about their previous clinical placements. Students were then divided into groups of 5 or 6, prioritizing diversity of previous clinical experiences. Each student group was assigned a supervisor responsible for leading group activities. The digital alternative included two main small-group activities: (1) clinical case studies and (2) digital lectures with webinars. In addition, students were to conduct a physiotherapy assessment of a family member or housemate and to summarize the findings in an anonymized medical record. the intervention lasted for 5 weeks. | NA | knowledge, skills and general competence: evaluation at the end of clinical placement period; reflection on their learning process during the online teaching period: final year written assignement (thematic analysis) | two main themes: (1) benefits and challenges of online alternative placement, (2) the importance of educational activities for achieving learning objectives. Online alternative placement seemed to provide valuable learning and experiences. Peer-to-peer discussions of a wide variety of clinical cases taken from different practice arenas appeared to be especially valued. The fact that students had to take main responsibility for preparing and leading the seminars was an important pedagogical aspect of the online alternative. | yes |

Table I: individual sources of evidence. NI = no information, NA = not applicable
