# Supplementary material for: Overview of styles, content, learning effects and attitudes of students towards digitally enhanced physiotherapy education – a scoping review
Source: BMC Med Educ. 2025 Feb 4;25:176. doi: 10.1186/s12909-025-06750-6 (PMC11792568; doi:10.1186/s12909-025-06750-6)
Supplement: Supplementary file 2 — Supplementary Material 2 [file 12909_2025_6750_MOESM2_ESM.docx]

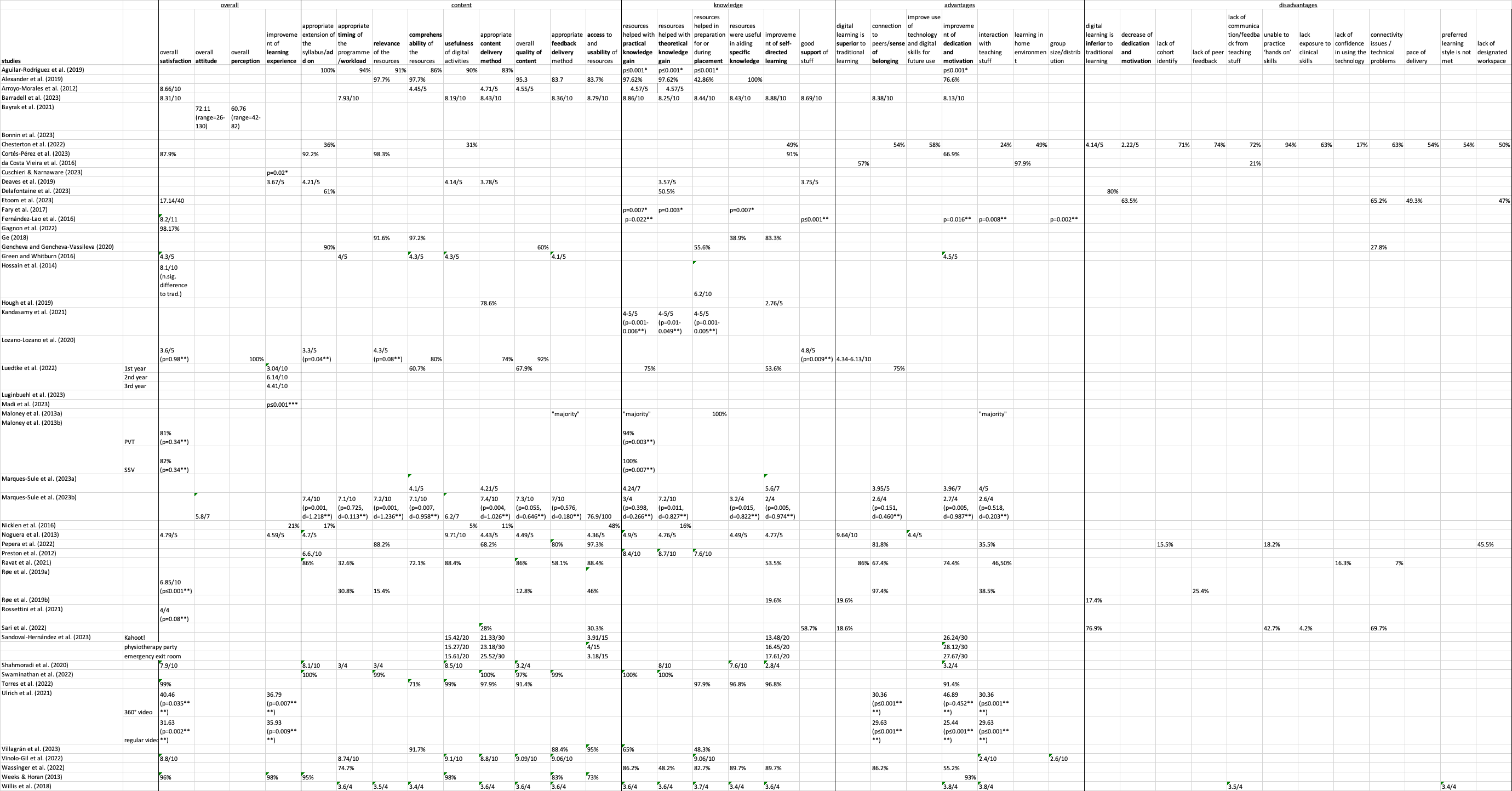


table II: summary of quantitative outcomes of students' satisfaction, attitude and perception (* improvement / difference baseline vs. after learning with digital content; ** difference compared to traditional learning in favor for the digital learning group; *** decrease / difference baseline vs. after learning with digital content; **** difference compared to traditional learning in favor for the traditional learning group)
