# Supplementary material for: Overview of styles, content, learning effects and attitudes of students towards digitally enhanced physiotherapy education – a scoping review
Source: BMC Med Educ. 2025 Feb 4;25:176. doi: 10.1186/s12909-025-06750-6 (PMC11792568; doi:10.1186/s12909-025-06750-6)
Supplement: Supplementary file 3 — Supplementary Material 3 [file 12909_2025_6750_MOESM3_ESM.docx]

|  | virtual learning environment | online syllabus | case scenario/ decriptive part | blog | power point | screencasts | videos | 2D/3D pictures | podcast/ audio | augmented reality | virtual reality | high fidelity mannequin | quizzes | escape room | physiotherapy party |
| --- | --- | --- | --- | --- | --- | --- | --- | --- | --- | --- | --- | --- | --- | --- | --- |
| Aguilar-Rodriguez et al. (2019) |  | x |  |  |  |  |  |  |  |  |  |  |  |  |  |
| Alexander et al. (2019) | King's eLearning and Teaching Service |  |  |  | x | x |  |  |  |  |  |  | x |  |  |
| Arienti et al. (2020) |  |  |  | S4BE |  |  |  |  |  |  |  |  |  |  |  |
| Arroyo-Morales et al. (2012) | ECOFISIO |  |  |  |  |  | x | x |  |  |  |  |  |  |  |
| Barradell et al. (2023) | x |  | x |  |  |  | x | x |  |  |  |  |  |  |  |
| Bayrak et al. (2021) |  |  |  |  |  |  |  |  |  |  |  |  |  |  |  |
| Bonnin et al. (2023) |  |  | x |  |  |  |  |  |  |  | x |  |  |  |  |
| Chesterton et al. (2022) |  |  |  |  |  |  |  |  |  |  |  |  |  |  |  |
| Cortés-Pérez et al. (2023) |  |  |  |  |  |  |  |  |  |  |  |  | Kahoot! |  |  |
| da Costa Vieira et al. (2016) |  |  |  |  |  |  |  |  |  |  |  |  |  |  |  |
| Cuschieri & Narnaware (2023) |  |  |  |  |  |  |  |  |  |  |  |  | Kahoot! |  |  |
| Deaves et al. (2019) |  |  |  |  |  |  |  |  |  |  |  |  |  |  |  |
| Delafontaine et al. (2023) |  |  |  |  |  |  |  | x |  |  |  |  |  |  |  |
| Etoom et al. (2023) |  |  |  |  |  |  |  |  |  |  |  |  |  |  |  |
| Fary et al. (2017) | RAP-eL |  | x |  |  |  |  |  |  |  |  |  |  |  |  |
| Fernández-Carnero et al. (2020) |  |  |  |  |  |  | x |  |  |  |  |  |  |  |  |
| Fernández-Lao et al. (2016) |  |  | x |  |  |  |  | x |  |  |  |  |  |  |  |
| Ferrer Sargues et al. (2021) |  |  |  |  |  |  | x |  |  |  |  | x | x | x |  |
| Gagnon et al. (2022) |  |  |  |  |  |  |  |  |  |  |  |  |  |  |  |
| Gardner et al. (2016) | RAP-eL |  |  |  |  |  |  |  |  |  |  |  |  |  |  |
| Ge (2018) | x |  | x |  |  |  | x |  |  |  |  |  | x |  |  |
| Gencheva and Gencheva-Vassileva (2020) |  |  |  |  |  |  |  |  |  |  |  |  |  |  |  |
| Green et al. (2014) |  |  |  |  |  |  |  |  |  |  |  |  |  |  |  |
| Green and Whitburn (2016) |  |  |  |  |  |  | x |  |  |  |  |  | x |  |  |
| Green et al. (2017) |  |  |  |  |  |  | x |  |  |  |  |  | x |  |  |
| Hossain et al. (2014) | MOOC |  |  |  |  |  | x |  |  |  |  |  | x |  |  |
| Hough et al. (2019) | x |  |  |  |  |  |  |  |  |  |  | x |  |  |  |
| Hurst (2016) |  |  |  |  |  |  |  |  | x |  |  |  |  |  |  |
| Kandasamy et al. (2021) |  |  |  |  |  |  |  |  |  | x |  |  |  |  |  |
| Lackovic et al. (2016) |  |  |  |  |  |  |  |  |  |  |  |  |  |  |  |
| Lo et al. (2022) | x |  |  |  |  |  |  | x |  |  |  |  |  |  |  |
| Lozano-Lozano et al. (2020) | ECOFISIO |  | x |  |  |  | x | x |  |  |  |  |  |  |  |
| Luedtke et al. (2022) |  |  |  |  |  |  | x |  |  |  |  |  |  |  |  |
| Luginbuehl et al. (2023) |  |  |  |  |  |  | x |  |  |  |  |  |  |  |  |
| Madi et al. (2023) |  |  |  |  |  |  |  |  |  |  |  |  |  |  |  |
| Major et al. (2020) | x |  | x |  | x |  | x |  |  |  |  |  | x |  |  |
| Maloney et al. (2013a) |  |  |  |  |  |  | x |  |  |  |  |  |  |  |  |
| Maloney et al. (2013b) |  |  | x |  |  |  | x |  |  |  |  |  |  |  |  |
| Marques da Silva et al. (2012) | cursos on-line |  | x |  |  |  | x | x |  |  |  |  |  |  |  |
| Marques-Sule et al. (2023a) | LMS | x |  |  |  |  |  |  | x |  |  |  | x |  |  |
| Marques-Sule et al. (2023b) | LMS | x |  |  |  |  | x |  |  |  |  |  | x |  |  |
| Munro et al. (2018) |  |  |  |  |  |  |  |  |  |  |  |  |  |  |  |
| Ng et al. (2021) |  |  |  |  |  |  |  |  |  |  |  |  |  |  |  |
| Nicklen et al. (2016) |  |  |  |  |  |  |  |  |  |  |  |  |  |  |  |
| Noguera et al. (2013) |  |  |  |  |  |  |  | x |  |  |  |  |  |  |  |
| Ødegaard et al. (2023) |  |  |  |  |  |  |  |  |  |  |  |  |  |  |  |
| Pepera et al. (2022) |  |  |  |  |  |  |  |  |  |  |  |  |  |  |  |
| Preston et al. (2012) | Physiotherapy eSkills Training |  | x |  |  |  | x |  |  |  |  |  |  |  |  |
| Ravat et al. (2021) | LMS |  | x |  |  |  | x |  | x |  |  |  | x |  |  |
| Røe et al. (2019a) |  |  |  | x |  |  | x |  | x |  |  |  |  |  |  |
| Røe et al. (2019b) |  |  |  |  |  |  | x |  | x |  |  |  |  |  |  |
| Rossettini et al. (2021) |  |  |  |  |  |  |  |  |  |  |  |  |  |  |  |
| Ruiz-Ruiz et al. (2023) |  |  |  |  |  |  | x |  |  |  |  |  |  |  |  |
| Sari et al. (2022) |  |  |  |  |  |  |  |  |  |  |  |  |  |  |  |
| Sandoval-Hernández et al. (2023) |  |  |  |  |  |  |  |  |  |  |  |  | Kahoot! | x | x |
| Shahmoradi et al. (2020) |  |  |  |  |  |  |  |  |  |  | x |  |  |  |  |
| Soundy et al. (2021) |  |  |  |  | x |  |  |  |  |  |  |  |  |  |  |
| Swaminathan et al. (2022) | LMS |  | x |  |  |  | x |  |  |  |  |  | x |  |  |
| Torres et al. (2022) |  |  | x |  |  |  | x | x | x |  |  |  |  |  |  |
| Ulrich et al. (2021) |  |  |  |  |  |  |  |  |  |  | x |  |  |  |  |
| Villagrán et al. (2023) | C1DO1 |  |  |  |  |  | x |  | x |  |  |  |  |  |  |
| Vinolo-Gil et al. (2022) | Nearpod |  |  |  | x |  | x | x |  |  |  |  | x |  |  |
| Vrzic et al. (2022) |  |  |  |  |  |  |  |  |  |  |  |  |  |  |  |
| Wassinger et al. (2022) |  |  |  |  |  |  |  |  |  |  |  |  | x |  |  |
| Weeks & Horan (2013) |  |  |  |  |  |  | x |  |  |  |  |  |  |  |  |
| Willis et al. (2018) |  |  |  |  |  |  | x |  |  |  |  |  |  |  |  |
| Wojniusz et al. (2022) |  |  |  |  |  |  | x |  |  |  |  |  |  |  |  |

*Table II: content delivery methods across the individual sources of evidence*
